# Neurophotonics: a comprehensive review, current challenges and future trends

**DOI:** 10.3389/fnins.2024.1382341

**Published:** 2024-05-03

**Authors:** Beatriz Jacinto Barros, João P. S. Cunha

**Affiliations:** ^1^INESC TEC – Institute for Systems and Computer Engineering, Technology and Science, Porto, Portugal; ^2^Faculty of Engineering, University of Porto, Porto, Portugal

**Keywords:** neurophotonics, neuromodulation, neuroimaging, optogenetics, microfluidics

## Abstract

The human brain, with its vast network of billions of neurons and trillions of synapses (connections) between diverse cell types, remains one of the greatest mysteries in science and medicine. Despite extensive research, an understanding of the underlying mechanisms that drive normal behaviors and response to disease states is still limited. Advancement in the Neuroscience field and development of therapeutics for related pathologies requires innovative technologies that can provide a dynamic and systematic understanding of the interactions between neurons and neural circuits. In this work, we provide an up-to-date overview of the evolution of neurophotonic approaches in the last 10 years through a multi-source, literature analysis. From an initial corpus of 243 papers retrieved from Scopus, PubMed and WoS databases, we have followed the PRISMA approach to select 56 papers in the area. Following a full-text evaluation of these 56 scientific articles, six main areas of applied research were identified and discussed: (1) Advanced optogenetics, (2) Multimodal neural interfaces, (3) Innovative therapeutics, (4) Imaging devices and probes, (5) Remote operations, and (6) Microfluidic platforms. For each area, the main technologies selected are discussed according to the photonic principles applied, the neuroscience application evaluated and the more indicative results of efficiency and scientific potential. This detailed analysis is followed by an outlook of the main challenges tackled over the last 10 years in the Neurophotonics field, as well as the main technological advances regarding specificity, light delivery, multimodality, imaging, materials and system designs. We conclude with a discussion of considerable challenges for future innovation and translation in Neurophotonics, from light delivery within the brain to physical constraints and data management strategies.

## Introduction

The field of contemporary Neuroscience is engaged in exploring the nervous system at a multitude of scales, encompassing molecular and cellular, subsystems, and entire organism levels (Soe et al., 2012). Despite significant advances, several challenges remain. These include the complexity and heterogeneity of the brain, characterized by millions of interconnected neurons (Sim et al., 2017), the difficulty in studying its size and intricacy, the inaccessibility of parts located within the skull (Boyden, 2011), limited tools for *in vivo* study, and the high speed of operation, with electrical activity patterns relevant to behavior taking place at the millisecond scale (Gradinaru et al., 2007). Despite substantial progress in the study of the nervous system, the understanding of the mechanisms behind its natural behaviors and responses to disease states remains extremely limited. As a consequence, the prevalence and global burden of nervous system disorders – including Alzheimer’s Disease (AD), Parkinson’s, and epilepsy – continues to increase, affecting millions of people worldwide. Despite considerable advances in medical treatments, including pharmacotherapy and electrical stimulation devices, their effectiveness is often limited by non-specificity and the occurrence of side effects (Rivnay et al., 2017). Therefore, addressing these disorders is a key challenge in neuroengineering, medicine, and science. Advances in this field are contingent upon technological progress toward the development of innovative tools for cell-type specific manipulation and observation.

Neural engineering research has produced a multitude of electrical, optical, chemical, and genetic tools for examining and controlling neural activity with increasingly high temporal and spatial resolution (Chen et al., 2017). These efforts strive to uncover fundamental neural functions, by manipulating and recording neuronal activity *in vivo* (Sim et al., 2017). Electrical stimulation and electrode-mediated recording have been the gold-standard methodologies applied to investigate neural circuits since these provide outstanding sensitivity and temporal resolution. However, some important constraints include limited *in vivo* spatial resolution and lack of efficiency to target multiple cells (Emiliani et al., 2015). Light-based techniques present clear advantages for monitoring and modulating neural activity. They are non-invasive, allowing for precise and flexible targeting of specific groups of neurons, and are capable of performing multiple tasks through the use of different wavelengths (Emiliani et al., 2015). Besides, optical tools hold the potential to offer a high spatial and temporal resolution, facilitating simultaneous interaction with numerous neurons (Doronina-Amitonova et al., 2015). A remarkable revolutionary advance was the introduction of optogenetics that, through the use of light-sensitive proteins, allows the manipulation of neurons and circuits with groundbreaking sub-microsecond precision (Boyden, 2011; Emiliani et al., 2015). Recent powerful trends include technologies that integrate multiple modes of interaction with neurons into a single device and allow for bidirectional communication with neural circuits with greater spatiotemporal accuracy (Frank et al., 2019). These advancements are not only aiding at a more extensive understanding of the brain, spinal cord, and peripheral circuits in the context of health and disease but also guiding the development of future, closed-loop therapies for neurological conditions (Canales et al., 2018; Frank et al., 2019).

Optical technologies are, therefore, at the forefront of various informative approaches to brain research, including high-resolution nonlinear optical and chemically selective microscopy and the widely used fluorescent biomarkers (Doronina-Amitonova et al., 2015; Cohen and Farhi, 2018). The ability to control light in space and time has recently been merged with traditional neuroscience approaches, resulting in the field of neurophotonics – a rapidly evolving cross-disciplinary area of natural sciences. This field encompasses the development of cutting-edge tools that use light to precisely and non-invasively perform manipulation and monitoring of individual and/or multiple brain cells and synapses (Ruthazer et al., 2022), with a wide range of neurobiology applications, from brain functional diagnostics, stimulation of neural networks, and molecular engineering, toward effective and selective diagnosis and therapy solutions for neurodegenerative and psychiatric diseases (Doronina-Amitonova et al., 2015; Ruthazer et al., 2022).

This review is focused on discussing the progress of the last decade in neurophotonics research. The work presented here aims to systematize the advances in photonic tools and methodologies, as well as identify new research trends, challenges and potential directions in this promising area. From a total of 166 screened articles, 56 representative works from the past 10 years, depicting innovative photonic approaches applied to Neuroscience case studies, were carefully selected by a systematic methodology [PRISMA 2020 (Page et al., 2021)]. The discussion is organized into six categories, concerning the main applications identified: (1) Advanced optogenetics, (2) Multimodal neural interfaces, (3) Imaging devices and probes, (4) Targeted therapeutics, (5) Remote operations, and (6) Microfluidic platforms. For each topic, the main challenges and motivations are debated, followed by a discussion on purposed innovative solutions. We intend not to evaluate in full detail each technology selected, but rather provide a broad perspective on the recent state-of-the-art of Neurophotonics. Although other reviews on the topic already exist, they focus on specific brain areas (Suter et al., 2014) or merely on imaging operations (Abdelfattah et al., 2022). Besides, some were published over 7 years ago (Doronina-Amitonova et al., 2015; Cho et al., 2016). Therefore, this work complements the existing literature by providing not only diverse examples of the most recent, innovative and impactful technologies under the Neurophotonics umbrella, but also by comparing and analyzing the evolution of paradigms, techniques and materials used, the challenges that remain in need of innovative approaches (light delivery, physical constraints, data management) and an insightful reflection on future directions for effective innovation and translation in this promising area.

## Methodology

The main goal of the present review is to understand the advances in neurophotonics research, regarding methodologies, tools and tackled scientific problems. Preceding the systematic analysis conducted, the following research questions were selected as guidance: (1) What are the main photonic tools applied in the context of neuroscience research? (2) What are the main neuroscience challenges/problematics tackled by photonic technologies? and (3) What gaps exist and what challenges remain for future research? In order to select the relevant scientific articles for the proposed systematic review, a multi-source research was conducted using three major electronic databases: PubMed, Web of Science and Scopus. For each database, a similar set of keywords was applied, based on the terms “Neurophotonics,” “Photonic devices” and “Optics and Photonics” applied to the “Neuroscience” and “Brain” research fields. A more detailed description of the search terms and retrieved results for each database is presented in Table 1. Notes, letters, reviews and erratum were excluded. A temporal period of 10 years (2013 – present) was selected. Each database was last consulted in August 2023.

**Table 1:** Electronic databases used for the conducted review, with the corresponding search query applied and number of total retrieved results.

Database	Search query	Retrieved results
Scopus	“Neurophotonics” AND [Field] “Neuroscience” “Photonic devices” AND [Field] “Neuroscience” “Photonics” AND “Brain” AND [Field] “Neuroscience”	14 20 18
		Sub-total: 52
PubMed	[MeSH Term^1^] “Optics and Photonics” AND [MeSH Term] “Neuroscience” [Title/Abstract] “Neurophotonics” [Title/Abstract] “Photonic” AND [Title/Abstract] “Brain”	13 18 42
		Sub-total: 73
Web of Science	[Topic] “Neurophotonics” [Topic] “Photonic devices” AND [Topic] “Brain” [Topic] “Photonics” AND [Topic] “Brain”	29 46 43
		Sub-total: 118
		TOTAL = 243

**Figure 1:**
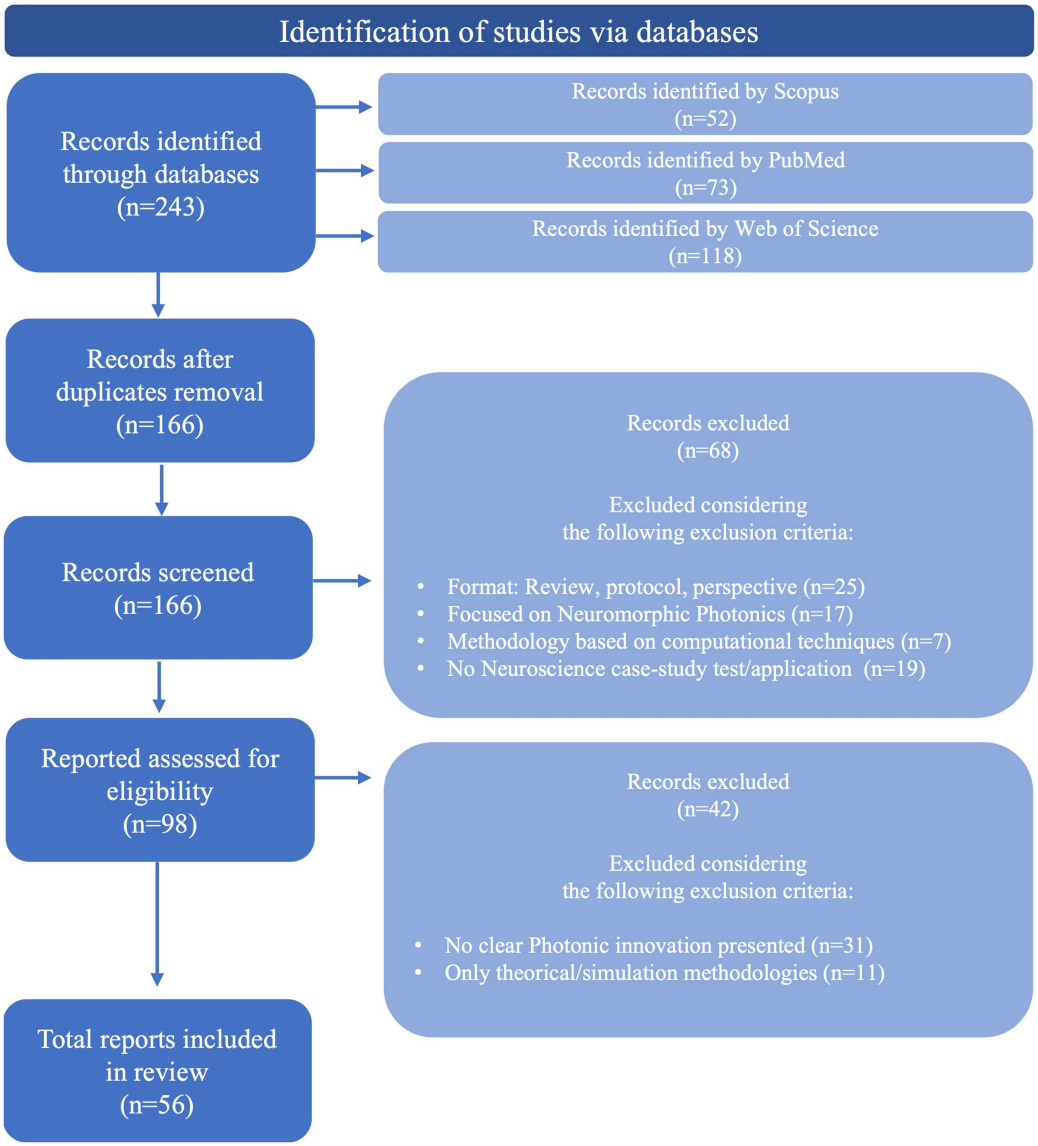
Methodology followed to select relevant studies from the retrieved results, based on PRISMA guidelines (Page et al., 2021).

The methodology followed to select the relevant studies from the retrieved results was based on the Preferred Reporting Items for Systematic reviews and Meta-Analyses (PRISMA) guidelines (Page et al., 2021), divided into three main steps, represented in Figure 1. The first step was the databases research presented above (Table 1), that retrieved a total of 243 references. A total of 77 duplicate records were removed in this step. Following that, 166 records were then screened by title and abstract. Articles presenting a methodology based on computational techniques, focused on photonic neuromorphic technology, a subject of computational sciences (use of neurophotonic paradigms for computational neural networks), and with no application or test conducted with a neuroscience case-study were excluded. Besides, articles in the format of reviews, perspectives, protocols and letters were also excluded. The remaining 98 articles were then evaluated for eligibility through full-text reading, where 42 were excluded by either only presenting theoretically and simulation results or by not including a clear innovation in Photonic technologies and methods. These included studies based on widely used microscopy tools, evaluation of physiological effects, case-studies, additional tests or small improvements to existing technologies. In case of encountering more than one study of the same group on a new technology, the paper where the method was completely described and properly tested for the first time was selected. The remaining 56 articles were considered for the intended discussion of recent advances in Neurophotonics, with the following information extracted: Year, photonic technology applied, brain target, aim and operations performed (sensing, imaging/recording, stimulation and optogenetics). The corresponding extracted data is synthesized and organized in Table 2. This methodology applies a restrictive selection process by considering simultaneously a moderately reduced timeframe (last 10 years), a clearly stated and fundamented photonic innovation, applied in a Neuroscience case study. This justifies the high number of records excluded. However, every requirement is essential to focus on the motivation questions stated previously and assure an accurate report of the most recent, innovative and impactful research in Neurophotonics.

**Table 2:** Results table obtained after the application of the complete literature search methodology.

Year	Photonic technology	Brain target	Aim	Operations				
				Sensing	Imaging/Recording	Stimulation	Optogenetics	Ref.
Advanced optogenetics								
2013	Patterned Stimulation	Retinal ganglion cells	*In vivo* holographic patterned stimulation	_	Fluorescence imaging; multielectrode array	Holographic light patterns	Chr2-eyfp	Reutsky-Gefen et al. (2013)
2014	High Power LEDs	Neural tissues	LED powered patterned optical stimulation	_	_	High power LED arrays	–	Cai et al. (2014)
2015	Tapered Optical Fibers	Mouse motor Cortex	*In vivo* multi-site fiber-based stimulation	_	Fluorescence Microelectrodes	Laser light	–	Pisanello et al. (2015)
2017	Silicon-based photonic probes	Mice Cortex and Hippocampus	Delivery of complex illumination patterns	–	2P imaging Extracellular recordings	E-pixel illumination	ChR2 and GCaMP6s	Segev et al. (2017)
2017	Upconversion nanoparticles	Rodent brain tissues neurons	Upconversion optogenetic stimulation	_	Patch-clamp MEAs Fluorescence	NIR illumination	ChR2 or C1V1	Lin et al. (2017)
2018	Upconversion nanoparticles	Mouse ventral tegmental area	NIR UCNP-mediated deep brain optogenetics	Fast-scan cyclic voltammetry	Fiber-mediated recording	NIR pulses	ChR2	Chen et al. (2018)
2018	Fiber-Optic Interface	Adult zebra finches	Microfiber bundles for deep-brain light delivery	–	Two-channel fluorescence imaging	Fiber- light	_	Nathan Perkins et al. (2018)
2018	Biodegradable neural interface	Deep-brain of living mice	Intracranial Light Delivery and Detection	Fluorescence sensing	Fluorescence recording	Laser light	EGFP and oCHiEF	Fu et al. (2018)
2018	Multichannel Optrodes	Cochlea	Implant-based selective stimulation	–	Auditory responses via electrodes	Optical pulses	_	Xu et al. (2018)
2020	Reconfigurable Nanoprobe	Mouse visual cortex	*In vivo* selective neuron stimulation	_	Extracellular recording (Electrodes)	Spatio-temporal spike patterns	ChETA	Mohanty et al. (2020)
2022	Organic light-emitting diodes (OLEDs)	*Drosophila melanogaster* motoneurons	Multicolor optogenetic stimulation *in vivo*	_	Patch-clamp recordings	500 ms light pulses	BiPOLES-ChRmine	Ciccone et al. (2022)
Multimodal neural interfaces								
2014	Implantable optrodes	Mouse visual córtex	Compact neural recording system	_	Extracelular recording headstage recording	External light sources	_	Chamanzar et al. (2014)
2017	Multifunctional polymer fiber probes	Mice medial córtex and hippocampus	Recording, stimulation and microfluidic delivery	–	gCPE electrodes	integrated PC/COC waveguides	ChR2	Park et al. (2017)
2018	Optoelectronic probes	Mouse motor cortex	*In vivo* stimulation and recording	–	MEAs	Laser light pulses	Chrimson+ group	Li et al. (2018)
2018	LED-based Photonic device	Brain Tissue	Monitor surface temperature	Probe temperature sensing	Microelectrodes	LED stimulation	–	Dehkhoda et al. (2018)
2018	Ultrathin Optrode	Anterior olfactory cortex	Localized stimulation and recording	_	Extracellular recording (electrodes)	Blue light (450 nm)	ChR2 (L132C/T159C)	Libbrecht et al. (2018)
2019	Bioresorbable optical sensor	Intracranial space	Monitor pressure and temperature	ICP and ICT	_	_	_	Shin et al. (2019)
2019	Bioresorbable optical sensor	Mice parietal lobe	Monitoring physiological parameters	Temperature, oxygenation and neural activity	_	_	_	Bai et al. (2019)
2020	Multimodal photonic probe	Rat neocortex hippocampus	Deliver IR light and measure cellular activity	Temperature sensing	Platinum recording sites	Continuous wave infrared light	_	Horváth et al. (2020)
2020	Fiber-optic thermometer	Mouse primary motor cortex	Brain temperature measurements	Temperature measurements	–	–	–	Fedotov et al. (2020)
2021	Neural optoelectrodes	Mouse cortex	On-demand, low-damage stimulation and neural activity readout	–	Neural Readouts (Electrodes)	Laser light	ChR2	Lanzio et al. (2021)
2022	Integrated “fibertrodes”	Mouse striatum and cortex	Fiber optogenetic activation	–	Extracellular recording of LFPs and AP	Laser light	ChR2	Spagnolo et al. (2022)
Targeted therapeutics								
2016	MNPs with NIR phototargeting	Astrocytes, Microglia cell lines	Therapeutics target, delivery and monitoring	Impedance sensing	CLSM imaging	NIR exposure	_	Sagar et al. (2016)
2016	Theranostic nanoparticles	Human Glioblastoma	Tracking of drug delivery and release	_	Luminescence monitoring	NIR Irradiation	_	Singh et al. (2016)
2016	Photonic crystal fiber	Human Glioblastoma	Multimodal multiphoton endomicroscope	_	Spectral and fluorescence measurements	Ultrashort light pulses	_	Ibrahim et al. (2016)
2017	Drug delivery platform	Adult mouse Cortical tissue	Delivery of therapeutic agents	–	Fluorescence microscopy	–	–	Son et al. (2017)
2018	Fiber theranostics	C6 Glioblastoma cell line	Phototheranostics deep brain tumors	–	Fluorescence imaging	Laser stimulation	–	Sharova et al. (2018)
2019	Upconverting nanoparticles	Microglia	In site activation of microglia	_	CLSM imaging	NIR irradiation	–	Zhou et al. (2020)
2020	Photonic crystal sensor	Oligoden-droglioma	RI-based detection of oligodendroglioma	RI sensor	_	_	_	Nouman et al. (2020)
2020	Photonic crystal fiber	Aβ peptide	Raman detection of amyloid β	Aβ detection based on FERS	_	_	_	Eravuchira et al. (2020)
2020	Carbon dots	Coronal sections Neural cells Aβ peptide	Targeting and suppression of Aβ species	ELISA analyses	Fluorescence imaging	Red LED irradiation	_	Chung et al. (2020)
2021	Silicon Quantum Sheets	Orthotopic brain tumors	Target, inhibit and monitor brain tumors	_	Photoacoustic,magnetic resonance and bioluminescence	NIR illumination	_	Miao et al. (2021)
2022	Implantable Fiber Probes	Rat Stroke Model	Recording hydrogen peroxide and pH transients in brain	FluorescentProtein sensors: levels of pH and H_2_O_2_	Fluorescence Imaging	LED stimulation	SypHer3s and HyPer7	Pochechuev et al. (2022)
2022	Theranostic Nanoparticles	Glioblastoma Multiforme tumor	Photothermal therapy	_	Bioluminescence and NIR imaging	–	–	Wang et al. (2022)
Imaging devices and probes								
2013	Multimodal portable exoscope	Rat spinal cord (Sciatic nerves)	Minimally invasive *in-vivo* imaging of tissues	_	Two photon excitation fluorescence Second harmonic generation images	_	_	Smith et al. (2013)
2017	Miniaturized two-photon microscope	Postsynaptic Dendritic Spines	Brain imaging in freely behaving animals	_	FHIRM-TPM imaging	Laser pulses	GCaMP-6f	Zong et al. (2017)
2017	Miniature microscope	Songbird premotor cortex	Fluorescence imaging in freely behaving animals	_	Fluorescence imaging	Blue LED light	GCaMP6	Liberti et al. (2017)
2017	Fiber-bundle Probe	Transgenic mice somatosensory cortex	Three-dimensional optical readout from single neurons in live brain	_	Fluorescence imaging; confocal imaging	Fiber laser	EGFPs and YFPs	Fedotov et al. (2017)
2018	Optical-fiber neural interface	Mouse hypothalamus	Study of *in vivo* neuronal activity	Calcium reporter mediated sensing	Fluorescence imaging	Laser light pulses	GCaMP6	Simone et al. (2018)
2018	Two-photon imaging platform	Mouse brain cortex (Pyramidal neurons)	Map the positioning of fiber–single-neuron optical coupling	_	Two-photon imaging Fluorescence imaging	473-nm laser light	Thy1-EGFP	Pochechuev et al. (2018)
2018	Microphotonic needle probe	Transgenic mouse cerebral cortex	Sub-cellular endoscopic imaging	-	CMOS imaging	–	ArcCreERT2 x ChR2-EYFP	Tadayon et al. (2018)
2020	Near-infrared diffuse optical tomography	Motor cortex	Functional brain imaging via photonic tomography	oxy- and deoxy-Hemoglobin Concentration	Near-infrared optical tomography	–	_	Orive-Miguel et al. (2020)
2021	Implantable photonic neural probes	Mouse brain tissues	Deep brain imaging in freely moving animals	_	LSFM and calcium imagig	–	_	Chen et al. (2021)
2021	Parylene Implantable microimagers	Mouse brain slice	Minimally invasive endoscopic imaging	_	Waveguide array imager Fluorescence imaging	–	–	Reddy et al. (2021)
2021	Time-domain functional near-infrared spectroscopy	Brain tissues	Wearable and miniaturized fNIRS system	Hemodynamic responses: HbO and HbR	Near-infrared spectroscopy	–	–	Ban et al. (2022)
Remote operations								
2015	Optofluidic neural probe	VTA	Wireless drug delivery and photostimulation	–	–	μ -ILED array	ChR2-eYFP	Jeong et al. (2015)
2017	Upconversion micro-devices	Rat striatum, tegmental area, and visual cortex	Upconversion tetherless neural stimulation	–	Extracellular recording	NIR laser	ChR2 and C1V1	Wang et al. (2017)
2018	Upconversion nanoparticles	Mice secondary motor cortex	*In vivo* tetherless optogenetic inhibition	–	Extracellular recording	NIR illumination	eNpHR	Lin et al. (2018)
2018	LED-based device	Murine cancer model	Photodynamic theraphy	–	Fluorescence imaging	LED stimulation	–	Bansal et al. (2018)
2019	Optofluidic wireless neural probe	VTA; Dorsal hippocampus	Wireless drug delivery and photostimulation	–	–	μ-ILED array	ChR2	Zhang et al. (2019)
2019	Photoplethys-mographic Imaging	Rat parietal cortex	Contactless assessment of cerebral autoregulation	ECG and systemic arterial blood pressure	PPG video images	Visceral and Somatic	–	Lyubashina et al. (2019)
2020	Remote photonics	Human occipital lobe	Remote monitoring of hemodynamic changes	Multispectral nano-vibration	fNIRS	Visual checkerboard	_	Ozana et al. (2020)
2021	Nanoparticle Probe	Brain cortical region	Remote detection of bioelectric signals.	Electrophysiological signals	_	NIR light	_	Hardy et al. (2021)
2022	Remote photonic-based detection	Human cerebral cortex	Remote monitoring of nano-vibrations	Spatiotemporal analysis of speckle patterns	EEG and video-EEG Speckle patterns	_	_	Kalyuzhner et al. (2022)
Microfluidic technology								
2019	Multifunctional neural probe	Hippocampal CA3 and CA1 regions; Somatosensory cortex	Combined optical stimulation, recording and microfluidic drug delivery	-	Extracelular recordings (Electrode array)	Light pulses	Thy1-ChR2-YFP	Shin et al. (2019)
2023	Implantable photonic neural probe	Brain tissue	Integrating microfluidic structures onto neural probes	_	Fluorescence Imaging	UV/Near-UV light	_	Mu et al. (2023)

Scientific articles considered for discussion of recent advances in Neurophotonics are described regarding the year, photonic technology applied, brain target, aim and operations performed (sensing, imaging/recording, stimulation and optogenetics). Aβ, Amyloid β; AP, Action Potential; BiPOLES-ChRmine, Bimodal Optogenetic Excitatory-Controlled Chimeras; C1V1, Channelrhodopsin 1 Variant 1; Chr2, Channelrhodopsin 2; ChETA, Chimeric Channelrhodopsins Engineered for Two-Photon Activation; CLSM, Confocal Laser Scanning Microscopy; ECG, Electrocardiogram; EGFP, Enhanced Green Fluorescent Protein; eNpHR, Enhanced Halorhodopsin; EEG, Electroencephalogram; FERS, Fluorescence Emission Recovery Spectroscopy; fNIRS, Functional Near-Infrared Spectroscopy; GCaMP6, Genetically Encoded Calcium Indicator Version 6; HbO, Oxygenated Hemoglobin; HbR, Deoxygenated Hemoglobin; ICP, Intracranial Pressure; ICT, Intracranial Temperature; LED, Light-Emitting Diode; LFPs, Local Field Potentials; LSFM, Light Sheet Fluorescence Microscopy; MEAs, Microelectrode Arrays; MNPs, Magnetic Nanoparticles; NIR, Near-Infrared; OLEDs, Organic Light-Emitting Diodes; oCHiEF, Optogenetic Chimera for High-Resolution *In Vivo* Fluorescence Imaging; PPG, Photoplethysmogram; UCNP, Upconversion Nanoparticles; UV, Ultraviolet; VTA, Ventral Tegmental Area; YFPs, Yellow Fluorescent Proteins; μ-LED, Micro Light-Emitting Diode.

## Results

The corresponding extracted data is synthesized and organized in Table 2. Since Neurophotonics is a rapidly evolving cross-disciplinary area, several distinct applications are described in the 56 articles selected. In order to provide a complete overview of the field, such applications are used to segment the discussion and highlight the different technology innovations and corresponding challenges of each main neuroscience target operation. Therefore, results are categorized and discussed into six main areas: (1) advanced optogenetics, (2) multimodal neural interfaces, (3) innovative therapeutics, (4) imaging devices and probes, (5) remote operations, and (6) microfluidic platforms.

**Figure 2:**
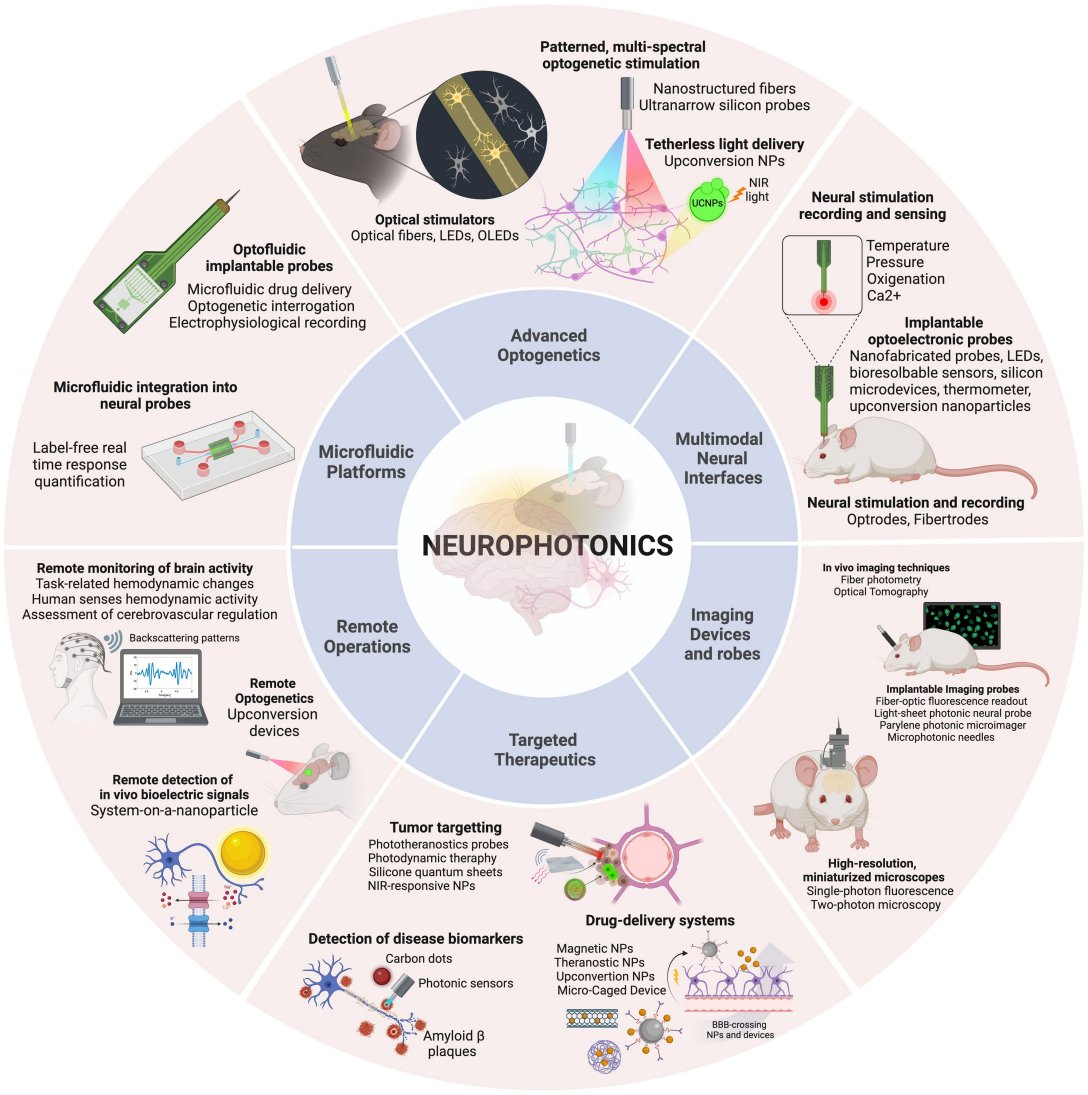
The discussion is organized into six categories, concerning the main applications identified: Advanced optogenetics, Multimodal neural interfaces, Imaging devices and probes, Targeted therapeutics, Remote operations, and Microfluidic platforms. Created with BioRender.com.

The 6 main areas of research, with the corresponding technologies included in this review, are presented in Figure 2 and will be detailed in specific sections of this review. Advanced optogenetics area comprises technologies that use optogenetic methods for stimulation of neuronal components. In this area the aim is to perform localized delivery of light for precise stimulation of the signaling molecules. In this section, different optical stimulators, patterned and multi-spectral solutions to deliver light, and new methods for tetherless light delivery will be discussed. In the Multimodal neural interfaces area, we aggregated platforms that integrate two or more operations – stimulation, recording and/or sensing – simultaneously. These include optrode and fibertrode designs, micro and nanophotonic implantable probes and new sensing mechanisms to monitor physiological parameters. In the Imaging devices and probes section, we present various technologies to observe brain activity, from low-cost, portable imaging devices to implantable imaging probes and photonic-based innovative imaging techniques. The Targeted therapeutics category contains technologies developed for three main goals: Tumor-targeting, drug-delivery and detection of disease biomarkers. A brief description of each main disease is provided, followed by a discussion of the different solutions presented for precise, efficient, and personalized therapies. The Remote operations area includes innovative technologies for tether-free optogenetics, wireless, miniaturized, and lightweight platforms to monitor brain activity and *in-vivo* detection of biosignals. Finally, in the category of Microfluidic Technology, different methodologies are discussed regarding photonic probe-embedded microfluidics for drug-delivery and microfluidic chip devices to study neural networks connectivity.

### Advanced optogenetics

**Figure 3:**
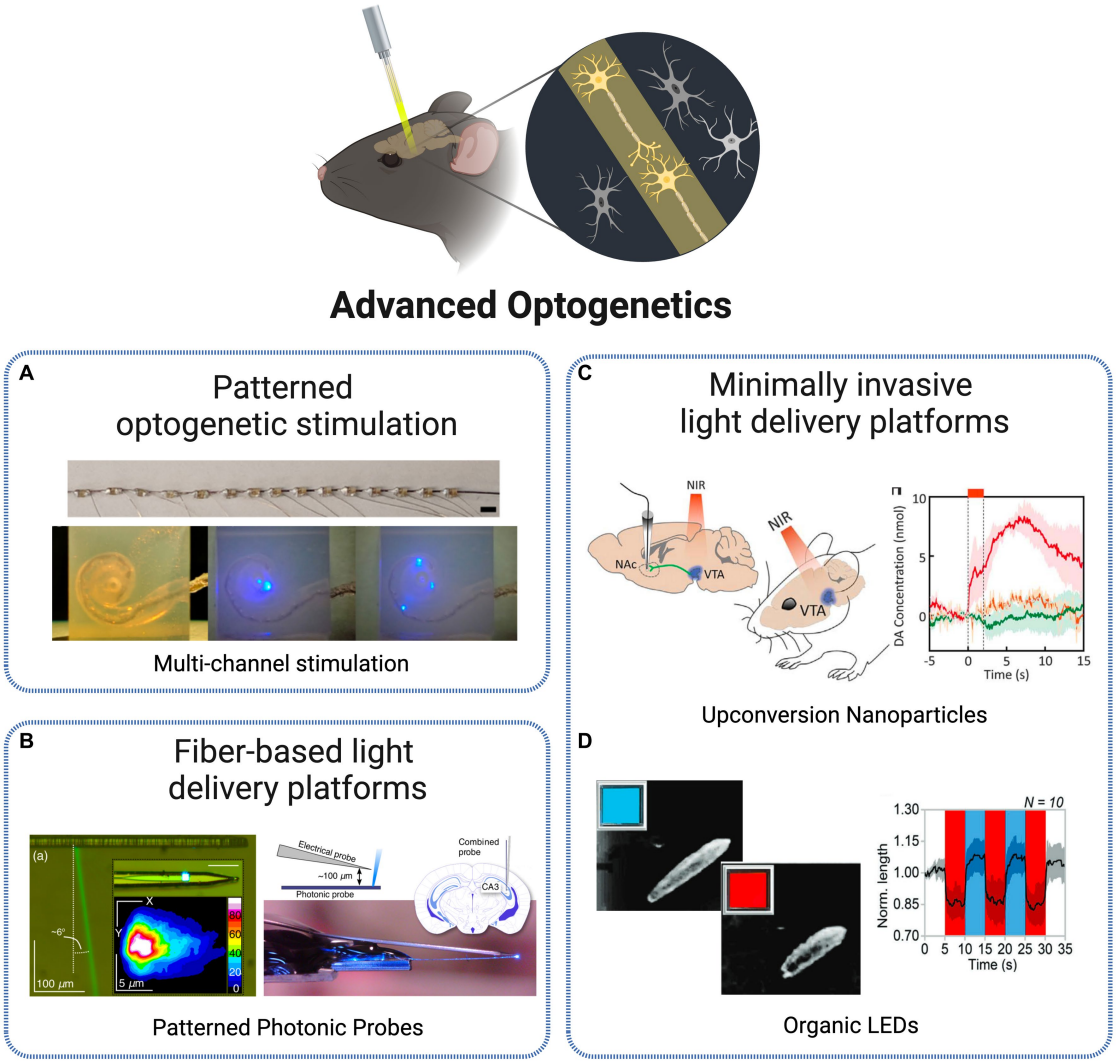
Technologies for optogenetic stimulation. **(A)** Multi-channel optrode-mediated stimulation constituted by an array of 15 μLEDs, inserted into a human scala tympani model (Xu et al., 2018). Licensed under CC BY 4.0. **(B)** Photonic probe with recording electrode tip and E-pixel optical stimulation (Segev et al., 2017). Licensed under CC BY 4.0. **(C)** *In vivo* transcranial NIR stimulation of the VTA using upconversion nanoparticles, with DA transient measurement in ventral striatum (Chen et al., 2018). Reprinted with permission from AAAS. **(D)** Blue-red OLEDs for Drosophila larvae motoneurons activation and inhibition (Ciccone et al., 2022). Licensed under CC BY 4.0. Created with BioRender.com.

The complexity of the brain, with billions of neurons highly interconnected through a sophisticated network of electrical and chemical signals, is a source of tremendous challenges in neuroscience research. Understanding the intricacies of this network is a major goal as it provides insights into the functioning of neuronal circuits and how they are related to the physiopathology of living systems (Yizhar et al., 2011). Recent advances in genetics and optics have revolutionized the investigation of functional connectivity in the brain by allowing simultaneous control and monitoring of neural activity. The combination of these two fields – known as optogenetics – has opened up new avenues for neuroscientists to study neural microcircuits and their functional connectivity in awake and behaving animal models (Deisseroth, 2011). The technique involves expressing light-sensitive proteins such as microbial opsins in neurons, intracellular organelles and molecules, performing labeling of the target structures, that can then be controlled by illumination (Boyden, 2011). The origin of optogenetics was prompted by the discovery of light-gated channels in the form of channelrhodopsins ChR1 (Nagel et al., 2002) and ChR2 (Nagel et al., 2003) and the application of ChR2 for exciting mammalian neurons with light (Boyden et al., 2005; Li et al., 2005). Since this revolutionary approach, optogenetics has become a versatile tool for precise manipulation of brain circuits, with the research efforts of several interdisciplinary groups originating the diverse toolbox of optogenetic tools available nowadays. Examples of advanced optogenetic stimulation techniques included in this study are presented in Figure 3.

One of the first critical aspects limiting optogenetic research was the ability to achieve control at a single-cell level. By having precise control over individual neurons, researchers could investigate the role of specific cells within a circuit and gain a better understanding of how circuits function. Significant progress has been continually made in the development of excitatory and inhibitory opsins that allow for direct optical control of cellular processes (Deisseroth, 2015). In order to achieve higher levels of precision in optogenetic tools, several complementary methods for delivering light with cellular specificity *in vivo* were proposed over the last decade. The initial conventional approaches for optical excitation of genetically targeted populations were based on light delivery tools such as optical fiber coupled illumination, LEDs and μ-LEDs, digital micromirror arrays or wide-field flashes, that deliver light directed to the entire population rather than in a specific manner (Reutsky-Gefen et al., 2013).

One solution adopted to tackle single-cell specificity was the use of patterned optogenetic stimulation. Reutsky-Gefen et al. demonstrated the first example of using a holographic pattern for optogenetic stimulation (Reutsky-Gefen et al., 2013). With this method, retinal ganglion cells expressing ChR2-eYFP were stimulated *in vivo* with a temporal precision in the millisecond timescale, with high cellular resolution through parallel multi-point stimulation delivered by the complex pattern. Another approach presents a patterned stimulation strategy based on high-power LEDs, where each frame can display flexible stimulation patterns (Cai et al., 2014). The 1,024 channels optical stimulation platform offers the possibility to manipulate the light delivery angle in each individual LED, producing flexible stimulation patterns refreshed at above 20 Hz. In Xu et al. (2018) present different methodologies to create multichannel optrodes with arrays of small optical sources such as side-emitting laser diodes (SELDs), vertical cavity surface emitting lasers (VCSELs) and μLEDs, that allow spatially selective stimulation through infrared neural stimulation. As an example, the authors depict a flexible optrode made with 15 connected μLEDs and show its potential to be applied in cochlear implants by a successful implantation into a human scala tympani model (Figure 3A). Another approach, at a smaller scale, is presented in Mohanty et al. (2020) based on nanophotonic optical technology. The authors present an implantable, 8-beam silicon-based nanoprobe, capable to stimulate specific sets of neurons with exceptional precision and simultaneously record their activity. This was achieved through a nanoscale switching network within the probe, controlling the flow of light dynamically. They demonstrated the ability to generate various neuron spike patterns *in vivo*, including sequential bursts and random pulses, all with sub-millisecond temporal precision. Their experiments highlighted the probe’s high-speed and reliable switching capabilities, suggesting its potential for precise control of neuronal populations.

Despite the advances in manipulating the transmission of light, important limitations arise at the time of its delivery to the brain. Optical stimulation and detection techniques, to be applied in the deep neural tissue, need to cope with scattering and absorption effects, demanding the use of relatively large optical probes and fibers to be implanted, which in turn cause significant damage to the tissue (Szarowski et al., 2003). Initial light delivery probe technologies such as fiber-based devices or large chip shank-like designs are highly effective in delivering light to deep tissues. However, these are characterized by large cross-sections that lead to tissue destruction, activating immunological response and disrupting the biological system. Multiphoton stimulation and microfabricated multi-site light delivery probes are among the most notable methods attempting to address invasiveness issues while achieving deep tissue light delivery. These methods aim to reduce tissue damage and immunological response while maintaining high precision and resolution, paving the way for non-destructive and minimally invasive optical stimulation and detection in deep tissue (Fain et al., 2017).

Pisanello et al. (2015) elaborated a technology tackling such challenges, through the use of tapered, nanostructured optical fibers as light delivery platforms for optical control of neural activity *in vivo*. The properties of the fibers – flexibility, small size and high spatial resolution – allow for a minimally invasive operation within the brain in a highly controlled manner. The authors present an advanced multi-site optogenetic stimulation achieved through the ability to switch, along the length of the taper, optical excitation to different regions of neural tissue. This allows for dynamic and selective illumination of different regions, providing optogenetic control of multiple brain sites. The device was tested in combination with MEAs-mediated extracellular recording. When tested in awake mice, it showed a remarkable *in vivo* suitability for layer-selective optical control of neural activity.

Besides optical fibers, silicon has become over the years a suitable and conventionally selected material for optical neural implants due to a range of beneficial characteristics including optical transparency, mature fabrication methods, cost-effectiveness, high stability and reduced loss in biological environments (Fu et al., 2018). Several approaches were thus developed centered on this material for implantable photonic devices for deep-brain complex light delivery. One example is proposed in Segev et al. (2017), based on ultranarrow probes with embedded silicon-based nanophotonic components for the delivery of complex illumination patterns within brain tissues (Figure 3B). Local optogenetic neural activation was observed in the cortex of mice expressing GCaMP6, through both extracellular electrical recordings and two-photon functional imaging.

Another trending solution over the recent years is the miniaturization of tools used to deliver, manipulate and collect light within deep brain regions. The use of microfibers is an example of such paradigm. In Nathan Perkins et al. (2018), the authors suggest the implantation of numerous (hundreds to thousands) 8 μm multimode optical microfibers into the brain, as stimulation light sources. As the implanted fibers spread gradually through the brain, a detailed surface fluorescence image is obtained for the deep 3D neuronal volume. With this approach, optical delivery is extended to targeted deeper regions of the brain while tissue displacement, immunological response and local network disruption are minimized due to the small diameter of the fibers. Extending the miniaturization to an even lower scale, Lin et al. (2017) suggest an approach based on a specific type of nanoparticles designated UCNPs. The authors designed an implantable optrode based on UCNPs with the goal to achieve remote activation of brain tissues. The nanoparticles, based on NaYF4, emit visible light as a response to tissue penetrating NIR light. As an outcome, neurons expressing different ChR proteins (ChR2 or C1V1), when close or in direct contact with the nanoparticle, are stimulated. Another example is presented in Chen et al. (2018) where transcranial NIR UCNP-mediated optogenetics was applied to evoke dopamine release from genetically tagged neurons in the ventral tegmental area (Figure 3C). The activation of inhibitory neurons in the medial septum, silenced seizure by inhibition of hippocampal excitatory cells and triggered memory recall. This innovative strategy allows for light delivery deep within the brain, in a tetherless way and without requiring any type of electronic components, paving the way for new all-optical wireless optogenetic stimulation techniques able to control brain activity not only at the single-cell levels but also in a network perspective.

The research and implementation of new materials is resulting in the integration of very interesting properties into technologies for deep-brain optogenetic control. For instance, to address the compatibility and stability challenges of common silica fibers, biocompatible and biodegradable materials have been explored for the investigation of deep-brain neural activity. In Fu et al. (2018), PLLA fibers have been implemented as a biodegradable optical neural interface for intracranial light delivery and detection, enabling deep brain fluorescence sensing and optogenetic interrogation. The *in vivo* study involving fluorescence signal collection and optogenetic stimulation in freely moving animals demonstrates the feasibility of biodegradable photonic devices and systems for both fundamental biological studies and clinical applications within the human body. In device fabrication, an alternative to LEDs has been gaining popularity. Although commonly used as a light source for optogenetic experiments, these devices are characterized by a low biocompatibility, rigidity and bulk design, which prompted the interest for alternative OLEDs. These operate at a reduced voltage, allow fabrication on flexible substrates and offer more tunable optical properties. Ciccone et al. (2022) present an implementation and characterization of such devices for switchable multi-color emission. This property allows for bidirectional optogenetic control – the OLEDs are able to shift between blue and red/green light emission, triggering both optogenetic excitation and inhibition (Figure 3D). This operation was tested in ND7/23 cells and *Drosophila melanogaster* larvae expressing bidirectional optogenetic proteins, showing high capability for a precise control of neuronal activity through innovative bicolor optical brain stimulation *in vivo*. Another approach is presented in Hillebrandt et al. (2023) where authors suggest the use of ultra-high brightness OLEDs for application in vision loss and, more specifically, restoration of visual perception, by providing a technology able to perform high-resolution optogenetic control of parallel retinal cells able to be adapted and, in the future, implemented into light-amplifying prosthetics. Lastly, nanomaterials are a class holding great promise for neural activity research, by functioning as transducers for neural stimulation via thermal conversion or light upconversion, as implemented in the UCNPs-based implantable optrode discussed previously.

### Multimodal neural interfaces

**Figure 4:**
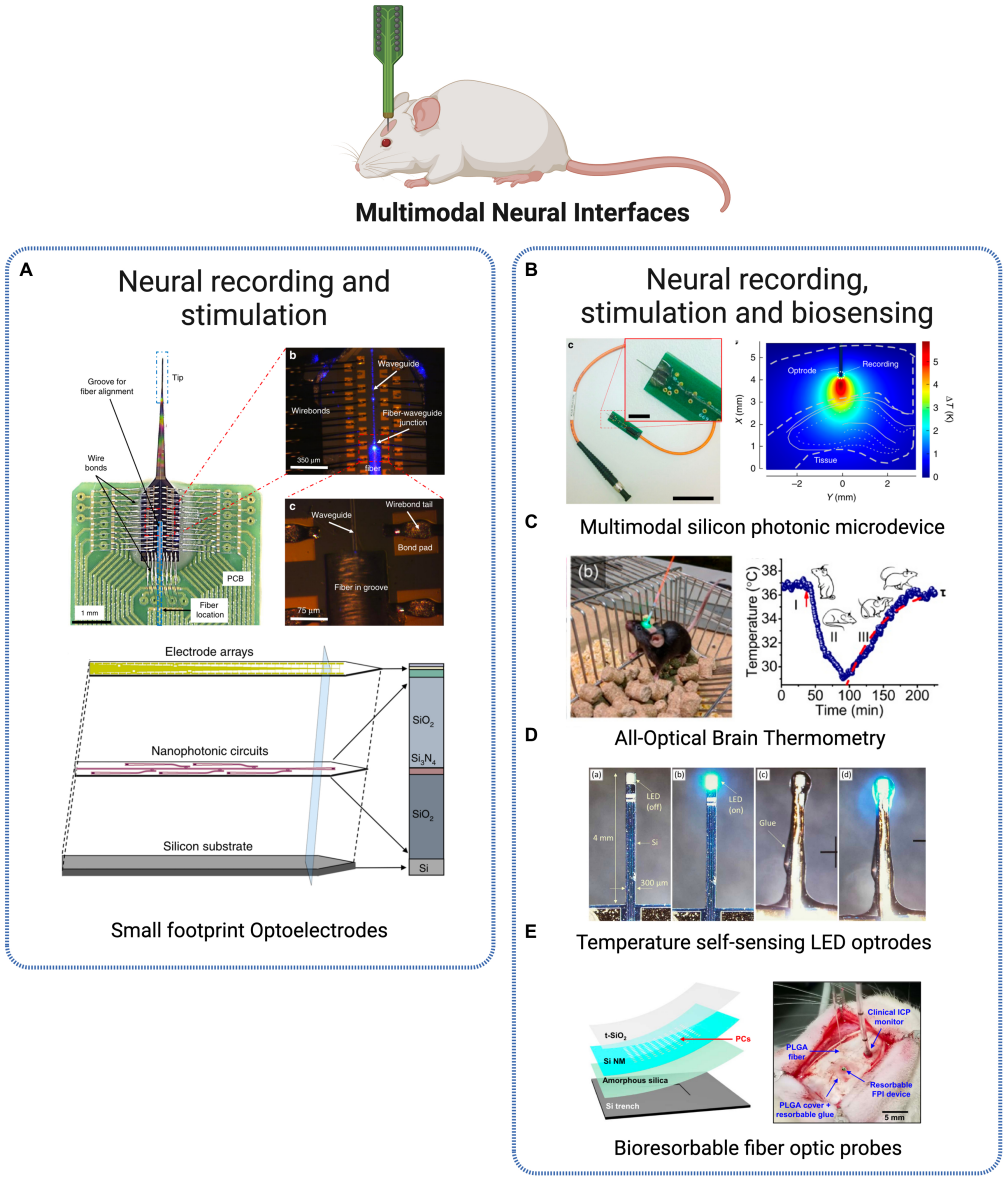
Multimodal Neural Interfaces. **(A)** Optoelectrode probe combining miniaturized arrays of sensors and nanophotonic circuits, combined for electrode-based neural readout and light delivery for optogenetic stimulation (Lanzio et al., 2021). Licensed under CC BY 4.0. **(B)** Silicon neural microprobe for infrared stimulation/inhibition and temperature sensing (Horváth et al., 2020). Licensed under CC BY 4.0. **(C)** Implanted fiber-optic thermometer in freely behaving mice (Fedotov et al., 2020). Reprinted with permission from American Chemical Society. Copyright 2020 American Chemical Society **(D)** LED-based optrodes mini-LED mounted on a silicon shank for temperature sensing (Dehkhoda et al., 2018). Licensed under CC BY 3.0. **(E)** Implantable bioresorbable sensor for pressure and temperature monitoring (Shin et al., 2019). Licensed under CC BY 4. Created with BioRender.com.

The demand for a multi-scale, mechanistic comprehension of brain function that accounts for local and whole-brain circuits remains a challenging task. One of the primary hurdles is the need for suitable tools to perform high-resolution monitoring of local neuron ensembles concurrently in various brain regions in awake and freely moving animals (Chamanzar et al., 2014). Given the crescent evidence that numerous cognitive behaviors implicate neural circuits spanned across multiple brain regions, the adoption of distributed recording and stimulation instruments is of utmost importance. A continuous challenge is the required trade-off between the range of stimulation and optical power, which ultimately results in an inherently low spatial resolution (Yizhar et al., 2011). This limitation directed research efforts toward the use of optical light guides to deliver light to target locations beneath the surface of the brain. More specifically, to enable light delivery and electrographic recording simultaneously from the intact central nervous system, researchers have designed dual optical and electrical probes, which are known as optrodes, combining both optical and electrical elements in a single device (Petrovic et al., 2022). The multimodal neural interfaces included and discussed are presented in Figure 4.

Optrodes were initially designed as a combination of commercial optical fibers and metal microelectrodes. Although this approach is relatively simple, it has its drawbacks, such as poor scalability, tissue damage when both fibers and electrodes are inserted in the same brain area, and limited control over the light emission geometry (Dufour and De Koninck, 2015). Over recent years, more advanced designs have emerged, featuring multiple sites for light delivery and electrical recording while attempting to minimize invasiveness and tissue damage. For such requirements, the use of micro and nanofabrication techniques is becoming a popular approach, providing new opportunities to produce compact, integrated and scalable optoelectronic probes. An example of microfabricated optrodes for high resolution electrophysiology and optogenetic stimulation is provided in Chamanzar et al. (2014). The authors describe the design and implementation of minimally invasive (50 μm × 20 μm) implantable optrodes with a silicon-parylene electrical layer for extracellular recording and an integrated photonic layer (microfabricated photonic polymer optical waveguides) for localized light delivery, with single-cell resolution. Another example is presented in Park et al. (2017) through a multifunctional polymer-based probe for opto-electrophysiological investigation of neural circuits in the mouse brain. The device includes an optical waveguide, six electrodes, and two microfluidic channels that allow for the injection of viral vectors carrying opsin genes, neural recording, and optical stimulation all in one. The miniature size of the device allows for multiple implantations in the brain, enabling investigations of brain circuits during behavioral experiments. The device composition – polymers and polymer composites – minimizes tissue response and allows for chronic, high-fidelity interrogation of brain circuits. Also focused on minimal size platforms, Libbrecht et al. present an ultrathin neural interface based on microchip technology, with 12 optical outputs (silicon nitride waveguides) and 24 electrodes (titanium nitride electrodes) (Libbrecht et al., 2018). The authors show the potential of the device for spatially confined optogenetic stimulation *in vivo*, measuring its effect in the anterior olfactory cortex of anesthetized and awake behaving mice. Due to the design of the device, it is possible to confine optical stimulation to small volumes with approximately single-cortical layer thickness, while recording simultaneously without measurable electrical artifacts.

Li et al. (2018) present an example of implantable optoelectronic probes manufactured using nanofabrication techniques for fully integrated electrical recording and optical stimulation. Electron beam lithography technique was used to fabricate 40 electrical recording and 6 optical stimulation sites within the optoelectronic probe. Nanofabrication offers an inherent advantage for device miniaturization and scalability, which are highly desired characteristics for the goal of scale up recording throughput and light delivery capabilities of optoelectronic probes. Examples of such type of fabrication are applied in Lanzio et al. (2021) and Lanzio et al. (2021) where the group presents advanced neural probes for electrode-based neural readout and light delivery for optogenetic stimulation, by combining micro- and nanofabrication techniques for high throughput and scalability. The authors present an innovative strategy based on the miniaturization of neural optoelectrodes by employing microfabrication techniques to create compact, versatile, and easily controllable neural probes (Figure 4A). These probes are designed with arrays of sensors and nanophotonic circuits that incorporate embedded ring resonators, thus creating a platform successfully brings together several desirable attributes for optoelectrodes, from reduced size and invasiveness, to expanded number of sensors and stimulation sites, while achieving precise light control without generating heat (Lanzio et al., 2021).

More recently, new approaches re-inventing and improving the optical fiber and electrodes pioneer combination of optoelectronic probes are rising. In Spagnolo et al. (2022), the authors introduce the concept of “fibertrodes” to describe the integration of microelectrodes on tapered optical fibers. Through microfabrication techniques (two-photon polymerization), multiple recording sides are fabricated around the tapered edge. Angled light delivery is used for optogenetic stimulation, while electrical recording is simultaneously performed in one up to three neurons, *in vivo*, with no photoelectric artifacts, demonstrating spatially confined optogenetic activation and simultaneous artifact-free extracellular recording of LFPs and action potentials.

Besides the stimulation and recording multimodal approaches presented above, a new optical dimension has been introduced into optical implantable devices – sensing of physiological parameters within the brain. One of the most commonly measured parameters is temperature, due to its relevance in tissue damage, cellular metabolism and response of neuronal populations in neurodegenerative disease stage development. In Horváth et al. (2020), authors present a multimodal photonic neural probe combining, beyond the optical and physiological operations, thermal sensing (Figure 4B). The three distinct functions are fully integrated into a single device, capable of performing electrical readout of individual cells at multiple locations along the probe shaft while simultaneously allowing temporal and spatial control of temperature within the deep-brain tissues. The highly multifunctional microsystem thus offers the possibility to measure cellular activity in deep neural tissues regarding thermally evoked responses.

For a similar purpose, another group presents an implantable, ultracompact thermometer based on optical fibers for high-resolution temperature measurements in the brain of freely behaving mice (Fedotov et al., 2020). Microcrystals coupled to the guided modes of a fiber-optic probe are used to generate a photoluminescence spectrum from which is possible to read out the local temperature within the brain (Figure 4C). The thermometer offers subcellular resolution with an accuracy within 0.15°C while also maintaining laser-induced brain heating below 0.1°C. Such approach presents a valuable alternative solution to invasive components such as thermocouples and thermoresistors for high-resolution brain temperature measurement *in vivo*. Beyond physiological processes, measurement and monitoring of temperature are also important when manipulating and introducing light delivery devices within the brain. Light sources require moderate to high-intensity irradiation at the target site, whose absorption can lead to localized tissue heating and damage. For this reason, a regulatory limit of 2°C is imposed to restrain the consequences of probe-derived temperature rising. In Dehkhoda et al. (2018), authors tackle precisely this challenge through the development of a method to calculate the surface temperature of implantable photonic devices. The approach is based on a LED, commonly used in optogenetic light delivery, capable of sensing and detecting changes in its temperature at the surface, thus preventing neural tissue damage actively during operation (Figure 4D). Another class of interesting multimodal tools to monitor physiological parameters is based on bioresorbable electronic sensors. Conventional implantable platforms require surgical removal after the operating period, with associated costs and risk of additional complications to the patient. Bioresorbable materials are thus being used for developing electronic technologies that dissolve at well-defined, programmable rates at the implantation site. Upon completion of the treatment, the bioresorbable technology will completely dissolve into harmless substances that are naturally cleared by the body, eliminating the need for additional surgeries. In Shin et al. (2019), the authors present a bioresorbable optical sensor for pressure and temperature monitoring and establish the feasibility of creating completely bioresorbable and Magnetic Resonance Imaging (MRI) compatible sensors (Figure 4E). Another approach introduces injectable multifunctional bioresorbable photonic devices for the analysis of biological tissues and fluids and continuous monitoring of critical physiological parameters such as tissue oxygenation, temperature, and neural activity, in freely moving animals (Bai et al., 2019). The device is designed for minimally invasive implantation and is constructed using materials that dissolve naturally through hydrolysis and metabolic clearance after a specified period of operation. Through continuous spectroscopic analysis, information on physiological status such as metabolic activity and tissue health is obtained from minimally invasive measurements into deep brain regions.

### Imaging devices and probes

**Figure 5:**
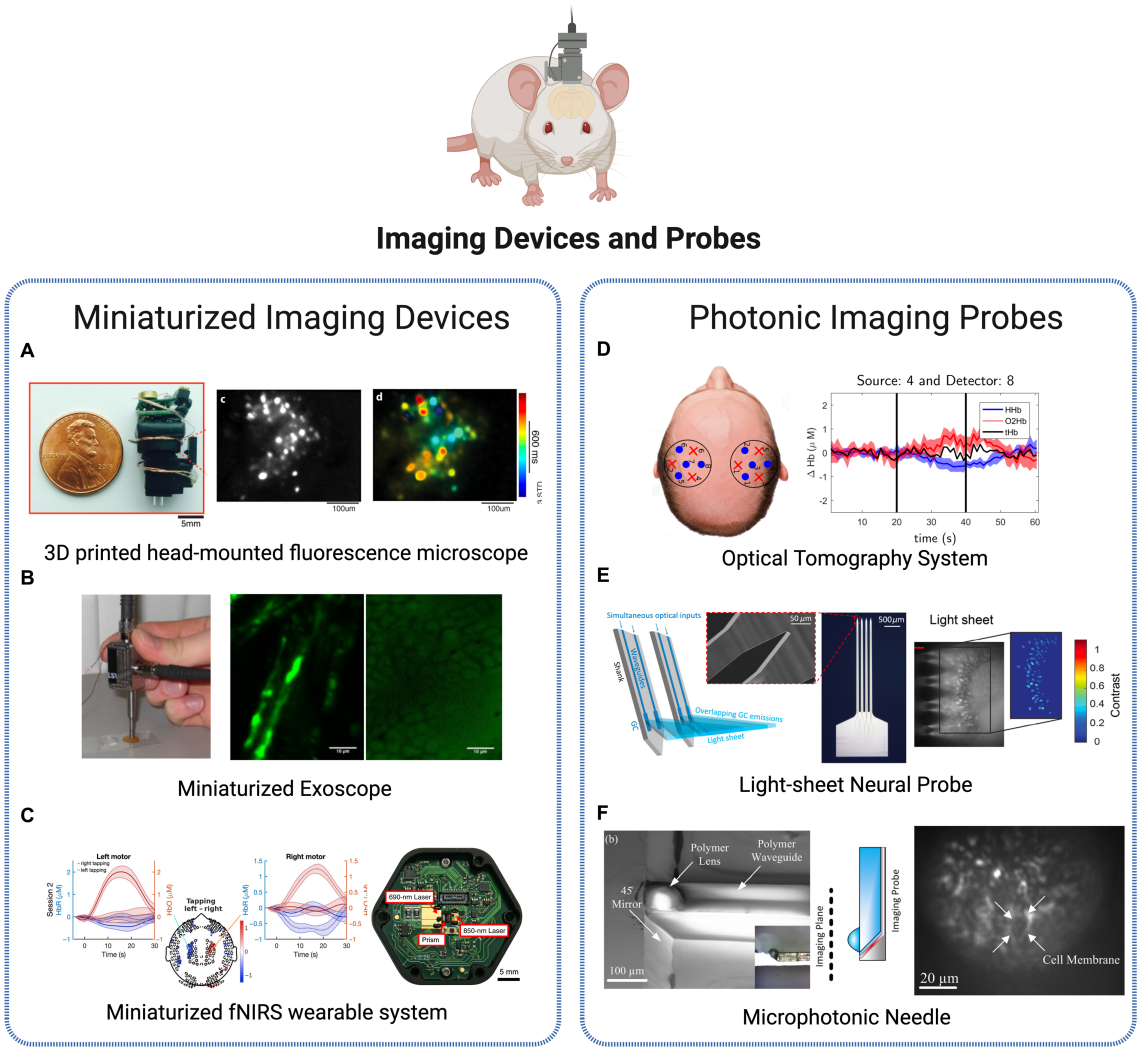
Brain imaging technologies. **(A)** Miniaturized imaging devices: 3D printed head-mounted fluorescence microscope with *in vivo* imaging (Liberti et al., 2017). Licensed under CC BY 3.0. **(B)** Portable, miniaturized exoscope for multimodal imaging of tissues. Images obtained demonstrating axons and surrounding fat cells in mouse sciatic nerve (Smith et al., 2013). Reprinted with permission of Optica Publishing Group. © Optical Society of America 2013. **(C)** Wearable time-domain fNIRS system offering whole-head covering through a miniaturized modular design. Brain activation is observed by measurement of hemodynamic changes (changes in the concentrations of HbO and HbR signals) in a finger tapping human experiment (Ban et al., 2022). Licensed under CC BY 4.0. **(D)** Diffuse Optical Tomography System for realtime oxygenated and deoxygenated hemoglobin concentration measurement (Orive-Miguel et al., 2020). Licensed under CC BY 4.0. **(E)** Light-sheet photonic neural probes for fluorescence imaging (Chen et al., 2021). Licensed under CC BY 4.0. **(F)** Microfabricated probe for sub-cellular level resolution endoscopic imaging (Tadayon et al., 2018). Licensed under CC BY 4.0. Created with BioRender.com.

The complexity of synaptic circuits is a great challenge to comprehend the brain functioning and information storage. Efforts to understand the connectome or the mapping of brain circuits have been made through mesoscopic methods such as electro-physiological analysis (Libbrecht et al., 2018) and functional image techniques (Bai et al., 2019; Shin et al., 2019). Fluorescence imaging in freely behaving animals has become a standard method to study neural circuit function, with miniaturized wide-field microscopes being applied for several years to observe brain activity while animals engage in voluntary behaviors. Several options are widely available, often characterized by low-cost, portable, and customizable designs and consumer-grade components. Technologies for deep-brain imaging are depicted in Figure 5.

Single-photon fluorescence imaging approaches, such as the system presented in Liberti et al. (2017), show to be a lightweight and inexpensive solution to longitudinal recordings of neural activity and structural dynamics in freely behaving animals (Figure 5A). More advanced designs as the two-photon high-resolution miniaturized microscope in Zong et al. (2017), now provide imaging of brain activity in freely moving animals at the single-dendritic-spine level, during extended periods of time, allowing the study of spatiotemporal dynamics in high detail and spatiotemporal resolution. Another application of two-photon imaging is presented in Pochechuev et al. (2018) where it is used to precisely determine the location of a fiber-optic probe in relation to individual neurons within the mouse brain cortex. This method allows for a detailed *in situ* assessment of the effectiveness of optical coupling between the fiber and individual neurons in the live brain of transgenic mice, which can support a selective stimulation and interrogation of individual neurons *in vivo* and be added to cranial-window imaging platforms and wearable miniature microscopes. Another application of *in vivo* imaging technology is presented by Smith et al. (2013) for minimally invasive imaging of tissues based on a multimodal, miniaturized exoscope. The device comprises a micro-electromechanical system scanning mirror and compact optics, and light delivery is achieved through the use of a photonic crystal fiber. The miniature objective lens is applied to observe the myelin surrounding the central axons in unstained, unfixed, fresh mouse sciatic nerves (Figure 5B). In Ban et al. (2022), authors present a wearable, whole-head coverage time-domain fNIRS where the innovative factor also relies on the miniaturization of components (Figure 5C). Contrary to the conventional large and complex NIRS systems, the suggested Kernel Flow device contains 52 small modules with dual wavelength (690 and 850 nm) laser source and a total of six detectors, mounted in a headset design that allows measurements over the frontal, parietal, temporal, and occipital cortices. Experiments based on finger-tapping task show the suitability of the system to accurately monitoring oxyhemoglobin and deoxyhemoglobin signals of human brain, with an equal or improved performance in comparison to traditional benchtop systems, thus representing a promising commercial approach for portable, miniaturized and non-invasive brain imaging.

The advances in fiber-optic components in Neuroscience have created numerous opportunities, including *in-vivo* brain imaging, functional studies of neurons in the brain, and the establishment of novel optical neural interfaces, through the control of neuronal activity through light. Additionally, the development of advanced fiber tools for high-speed fluorescence microscopy in freely moving animals has helped address long-standing challenges in the experimental study of the neuronal foundations of cognition and memory. A diversity of implantable microimagers have been purposed over recent years for functional brain imaging with cell-type specificity. Fedotov et al. (2017) presents a neurointerface composed of an optical fiber bundle for high-precision optical fluorescence readout and brain imaging formation. The analysis of individual fiber channels allows optogenetic recordings from single neurons at three dimensions, *in vivo*. In Simone et al. (2018), fibers are used to develop a low-cost photometry system to monitor *in vivo* calcium optical transients in neurons. The technique’s ability to measure calcium or other fluorescent indicators in target populations, coupled with its high-sensitivity recording, allows for the transformation of bulk activity interrogation into circuit-level neural encoding measurement. The customizable, low-light, fiber photometry system was applied in awake, freely moving mice to interrogate the behavioral correlates of the stress response. Another photonic-based imaging method – optical tomography – is also purposed using injectable fibers (silicon photomultipliers detectors) for dual-wavelength, real-time functional brain imaging (Orive-Miguel et al., 2020). The system allows to follow the brain activation variations over time through analysis of HbO and HbR concentration in the tissues (Figure 5D).

The research efforts are continuously converging to more flexible and compact implantable devices to reduce invasiveness and disruption in the brain. An innovative system presented in Chen et al. (2021) is the use of probe-enabled light-sheet fluorescence imaging. Implantable silicon photonic probes are able to generate multiple addressable light sheets in the targeted tissues at arbitrary brain depths, without requiring active micro-optical components on the probe, thus reducing the risk for tissue heating and displacement (Figure 5E). In Reddy et al. (2021), authors developed a micro-imager array with parylene photonic components for minimally invasive endoscopic brain imaging. The spatial resolution obtained show to be suitable for *in-vivo* fluorescence image studies, using the compact and minimalized device. Another example of a miniaturized, minimally invasive platform is provided in Tadayon et al. (2018) where authors describe a 100 μm cross-section probe for single cell resolution imaging (Figure 5F). The novelty of this work lies in the use of microfabrication methods (soft lithography, planar microfabrication) to fabricate 3D photonic elements (polymeric waveguide and polymeric micro-lens) with reduced cross-section while achieving high resolution. The potentialities of the probe were tested in combination with wide-field microscopy to image activated neural cells in brain slices, where neuron boundaries were clearly resolved at until 2 μm sized lithographic features. The authors present several options where the reduced size and sub-cellular resolution of these microphotonic needles can be a valuable advantage, namely minimally invasive imaging and excitation of deep brain tissues and diagnostics involving multiple modalities techniques emerging currently.

### Targeted therapeutics

**Figure 6:**
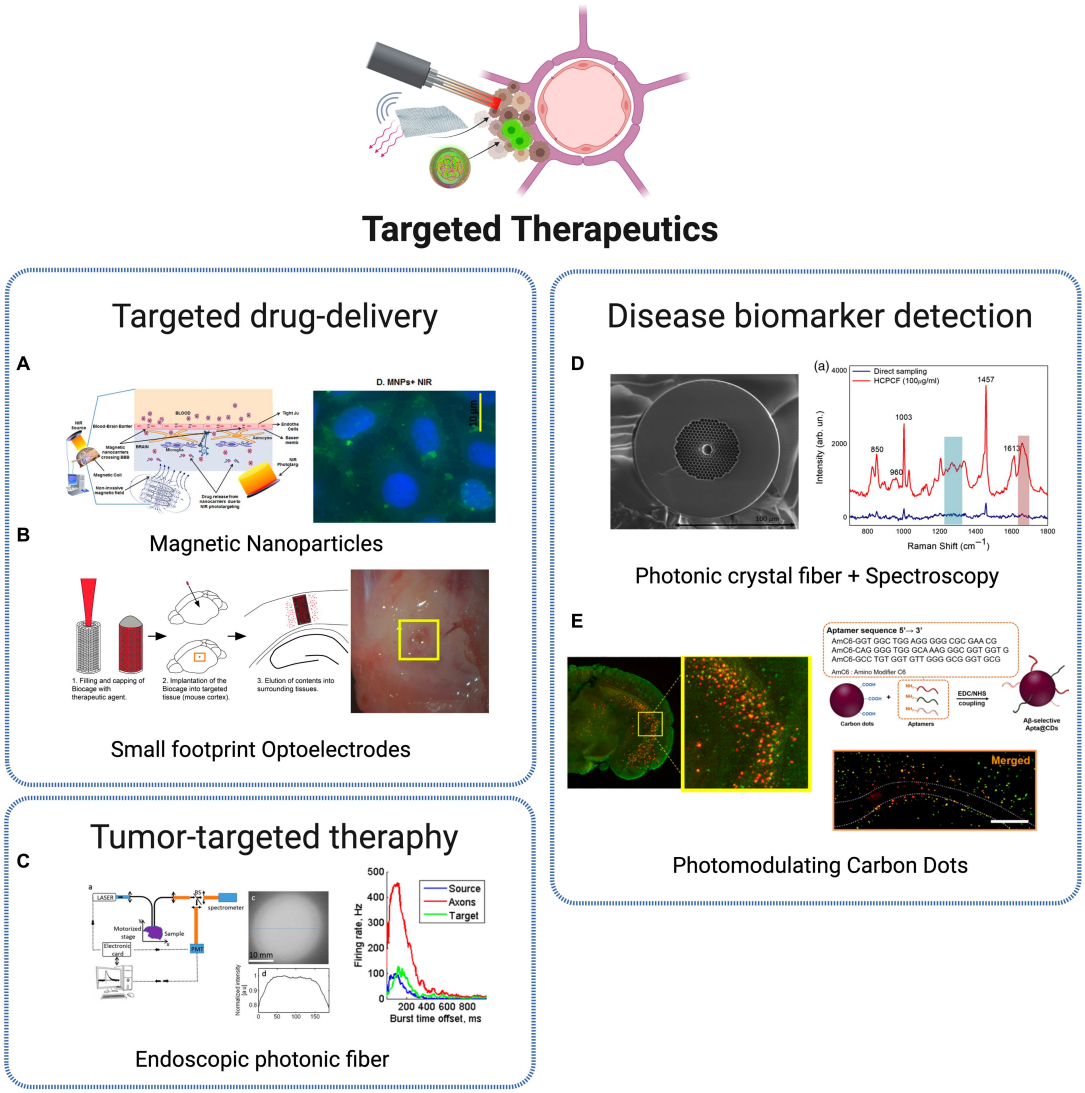
Targeted therapeutic technologies. **(A)** Non-invasive brain targeting for delivery and monitoring of therapeutics based on NIR phototargeting and Magnetic Nanoparticles (Sagar et al., 2016). Licensed under CC BY 4.0. **(B)** Implantable micro-caged device, implanted in a mouse cortex, for *in vivo* local delivery of agents (Son et al., 2017). Licensed under CC BY 4.0. **(C)** Photonic crystal fiber based endoscopic system, with excitation and fluorescence signal collection (Ibrahim et al., 2016). Reprinted with permission of Optica Publishing Group. © Optical Society of America 2016. **(D)** Photonic crystal fiber for AD biomarkers via Raman spectroscopy (Eravuchira et al., 2020). Licensed under CC BY 4.0. **(E)** Carbon Dots (CDs) for Alzheimer’s Aβ aggregation suppression by photomodulation (Chung et al., 2020). Reprinted with permission from Chung et al. (2020). Copyright 2020 American Chemical Society. Created with BioRender.com.

Modern medicine faces a significant obstacle in developing therapies that can achieve precision, efficiency and personalization for individual patients. In particular, the demand for technologies with modulation, therapeutical and monitoring capabilities to be applied in the brain is vast and diverse and achieving successful outcomes in this field remains a formidable task, with the majority of neuroscience diseases being highly dependent on therapeutical approaches with lack of specificity and side-effects. Innovative therapeutics discussed in this review are represented in Figure 6 regarding drug-delivery, tumor-targeting and biomarker detection applications.

The transcranial delivery of central nervous system (CNS) drugs is a large challenge for translating the advances in drug development research to clinical practice, mainly related to the presence of the blood–brain barrier (BBB), with the role to protect the brain from harmful substances while regulating the entry of essential nutrients. As a consequence, the efficiency of drug delivery is commonly low, which requires high doses of most CNS-targeted drugs, applied with reduced specificity, commonly leading to severe side effects in peripheral organs (Obermeier et al., 2013). Therefore, efficient BBB-crossing brain delivery systems are crucial to enhance therapeutic outcomes while avoiding adverse systemic effects. Several nanoparticle-based approaches have been explored for localized and specific drug-delivery systems. A promising and crescently used NPs class for the goal of non-invasive targeting and monitoring strategies are MNPs. Due to its superparamagnetic properties, it became possible to target specific cells and tissues through the local application of non-invasive magnetic forces. The well-structured and rigid architecture of MNPs provides a stable binding site for various diagnostic or therapeutic agents. Besides, external control over the movement of MNPs increases their ability to reach the target site, reducing their peripheral circulation time compared to other nanocarriers (Sagar et al., 2016). With proper dose control, MNPs should pose few safety concerns and are highly suitable for *in vivo* applications. It was recently demonstrated that such type of nanoparticles, coupled with NIR biophotonic light delivery systems, provides a promising and safe approach for non-invasive therapeutics, through controllable drug delivery within the brain (Sagar et al., 2016; Figure 6A). The recent advances in optics and photonics have led to the development of nanoparticles combining both diagnostic and therapeutic properties – the commonly known theranostic nanoparticles – that have been exploited with great results in therapy, real-time monitoring and diagnosis. One example was the development of theranostic photonic nanoparticles (TPNs), with biocompatibility and multifunctionality, able to efficiently cross the BBB and deliver a therapeutic agent, with simultaneous bioluminescence monitoring through the incorporated self-traceable properties (Singh et al., 2016). This was possible using self-assembled ultrasmall nanoparticles, integrated with photonic molecules. Such promising approach allowed the visualization *in vivo* of the drugs transcranial delivery and release, revealing a powerful translational potential for highly specific and personalized drug-delivery platforms. Another nanophotonic approach is based on a particular type of material showing high suitability for neuroscience applications, commonly referred to as upconversion material. This provides an advanced capability to convert low-energy photons such as NIR light, which can penetrate deep into biological tissues, into higher-energy photons such as visible or ultraviolet light, which can be used to excite neurons or other cells in the brain. This has tremendous potential for non-invasive or minimally invasive neural stimulation and imaging techniques. An example is presented in Zhou et al. (2020), where upconverting nanoparticles were used for controlled microglia activation in targeted brain regions. Microglia cells are essential components of the CNS and play a critical role in the immune response. However, to be effective, selective activation of these cells is necessary to avoid extensive pathologic neuroinflammation and neural damage. The BBB crossing, light-responsive nanoparticles developed are able to accumulate in the microglia cells and activate them in localized brain regions, through the local delivery of NIR light. For a therapeutical intervention, drugs are entrapped in the nanoparticles, thus the activity of macrophages is prevented, eliminating the problem of unintended activation in non-targeted areas of the brain. This concept for controlled microglia activation presents a promising translational potential for targeted microglia activation mediated therapy of various brain disorders, including severe infections, neurodegenerative diseases like Alzheimer’s, and cancer (Zhou et al., 2020). Beyond nanophotonic approaches, optical platforms for local drug delivery have been suggested for targeted delivery of therapeutic agents. One example is suggested in Son et al. (2017), named Biocage – a porous device containing molecules of interest that are released in a determined region over time. Using microfabrication techniques (3D laser lithography system), the micron-sized container provides a flexible, adaptable, and minimally invasive platform for precise and controlled delivery of direct therapies in tissues (Figure 6B).

Another major group of photonic-based therapeutic strategies presented in the last decade is focused on tumor-targeted therapy. Malignant brain tumors, particularly gliomas, are considered the most dangerous and devastating neoplasms due to their highly invasive nature. The excessive infiltration of glioma cells into healthy surrounding tissue poses a significant challenge to the efficacy of existing treatment approaches (Sharova et al., 2018). The current primary mode of treatment is surgical removal. However, glioma cells specific characteristics enable the tumor to escape complete resection, resulting in disease recurrence. The treatment of brain tumors is, therefore, a complex and multi-stage process, and there is a requirement for effective toolkits enabling multiple diagnostics and therapies for such neoplasms without the need for additional surgical intervention (Sharova et al., 2018). Conventional techniques have been improved for a more complete, precise and effective operation. Ibrahim et al. (2016) present a multi-photon endoscopic system with multimodal capabilities, through the introduction of customized small-core double-clad photonic crystal endoscopic fibers, capable of measuring spectral and lifetime data from endogenous fluorescence of freshly extracted human samples (Figure 6C). The photonic approach allows a reduced spectral acquisition time and beam power output compared to conventional optical fiber solutions for endoscopy applications. In Sharova et al. (2018), authors developed an intracranial implantable optical system for phototheranostics of deep-lying brain tumors. The platform is based on an optical fiber structure, within a neuro-scaffold, that is specifically placed into the tumor bed. The fluorescence signal is generated from malignant cells using photosensitive agents, which aids diagnosis and permanence growth monitoring through fiber optic probe with emitting and receiving fibers connected with a laser source and spectrometer, respectively. Interesting approaches have been also purposed using techniques based on nano agents, components and fabrication techniques, especially to target Glioblastoma multiforme (GBM), one of the most lethal and therapy-resistant tumors in the CNS. GBM is a primary brain tumor that is both common and lethal due to its poor prognosis, high mortality, and recurrence rates (Lim et al., 2018). Advanced surgery is the primary focus of GBM management, accompanied by radiotherapy and chemotherapy. However, surgery procedures are challenged by inaccurate tumor location, potential serious infection, and high craniotomy costs.

A photonic approach based on two-dimensional silicon quantum sheets (2D Si QSs) shows the potential of photonic nanoagents for orthotopic glioma theranostics (Miao et al., 2021). The Si QSs are fabricated through a scalable approach combining chemical delithiation and cryogenic exfoliation processes. The ultrasmall structures are able to cross the BBB and accumulate in glioma tissue, where the strong light-harvesting capability provides a platform for brain tumor ablation through photothermal therapy and growth management via photoacoustic imaging. Another nanoparticle-based theranostic approach was recently suggested, based on NIR IIb aggregation-induced-emission nanoparticles (Wang et al., 2022). These were used as platforms for theranostic GBM treatment by being combined with a brain-targeting peptide, that allowed to target the glioma and promote efficient BBB traversal. The biodistribution and accumulation of the nanoparticles in GBM tumor sites were monitored *in vivo* with fluorescence imaging, with the results showing an effective tumor ablation through induced heat transfer upon irradiation. A third group of photonic solutions is focused on the detection of disease biomarkers. Biophysical parameters and molecular biomarkers are precise indicators of a particular health or disease condition, and their identification is of great importance for the diagnosis, monitoring, and treatment of diseases. For instance, it has been established for several years a proportional relation of refractive index values with tumor malignancy degree (Biswas and Luu, 2009).

Nouman et al. (2020) used a simple one-dimensional photonic crystal to detect lesions in the brain exploring such principle. The sensing mechanism is based on resonant wavelength shifts when the refractive index of the brain lesion layer is altered. While different cells enter the photonic crystal cavity, the wavelength shifts of the resonant defect peak in the transmission spectra are calculated and used to detect different pathological conditions, such as GBM and Multiple sclerosis. Optical methodologies can also be valuable tools to measure and monitor transient disorder signatures against a high-floor noise background, as demonstrated in Pochechuev et al. (2022) where arrays of fiber-optic probes were used to record, in real-time, stroke-induced hydrogen peroxide and pH transients when implanted in ischemia-affected brain areas. The platform is able to provide optical excitation and fluorescence read out from genetically encoded fluorescent protein sensors, that are used to quantify stroke growth dynamics.

In AD, protein species such as Aβ peptide and tau protein are associated and recognized as main biomarkers. AD is recognized as one of the primary causes of dementia and is ranked as the fifth significant cause of mortality. The pathological features are distinguished by the buildup of extracellular plaques containing Aβ and the clumping of tau protein as intracellular neurofibrillary tangles in the brain. Given that Aβ peptide aggregates can exacerbate neuropathy and cognitive decline in AD patients, many attempts have been made to early detect and/or inhibit Aβ assembly as a potential management and treatment options for AD. Hollow core photonic crystal fibers were used in combination with conventional Raman technique to perform FERS measurements (Eravuchira et al., 2020) (Figure 6D). The low-cost, flexible technology allowed a label-free detection of amyloid β (1–42) peptide (Aβ42) with high sensitivity, using a reduced sampling volume. The photonic crystal components showed to be a suitable tool to obtain Raman signals of Aβ42, paving the way toward early detection of AD. A different approach is presented in Chung et al. (2020), where photonic nano agents – CDs – are used for *in vivo* photomodulation. CDs have gained attention due to their excellent biocompatibility, photophysical stability, and synthetic versatility compared to molecular dyes. Aβ-targeting, red-light-responsive nanosized carbon dots (∼5 nm) were produced to aid Aβ aggregation suppression *in vitro* and *in vivo*, with a spatiotemporal controlled light-nanomodulation approach (Figure 6E). CDs were functionalized for targeting capabilities toward Aβ42 species. When located, red LED irradiation was used to trigger functionalized CDs to irreversibly denature Aβ peptides. This way, Aβ aggregates formation was prevented, and Aβ-associated cytotoxicity was attenuated. The light-powered CD-mediated therapy lead up to a 40% reduction of Aβ burden at the targeted sites in the brain in comparison to nontreated brain hemispheres, which reveals their therapeutic capabilities as an effective nano-agent against abnormal amyloid burden *in vivo*.

### Remote operations

**Figure 7:**
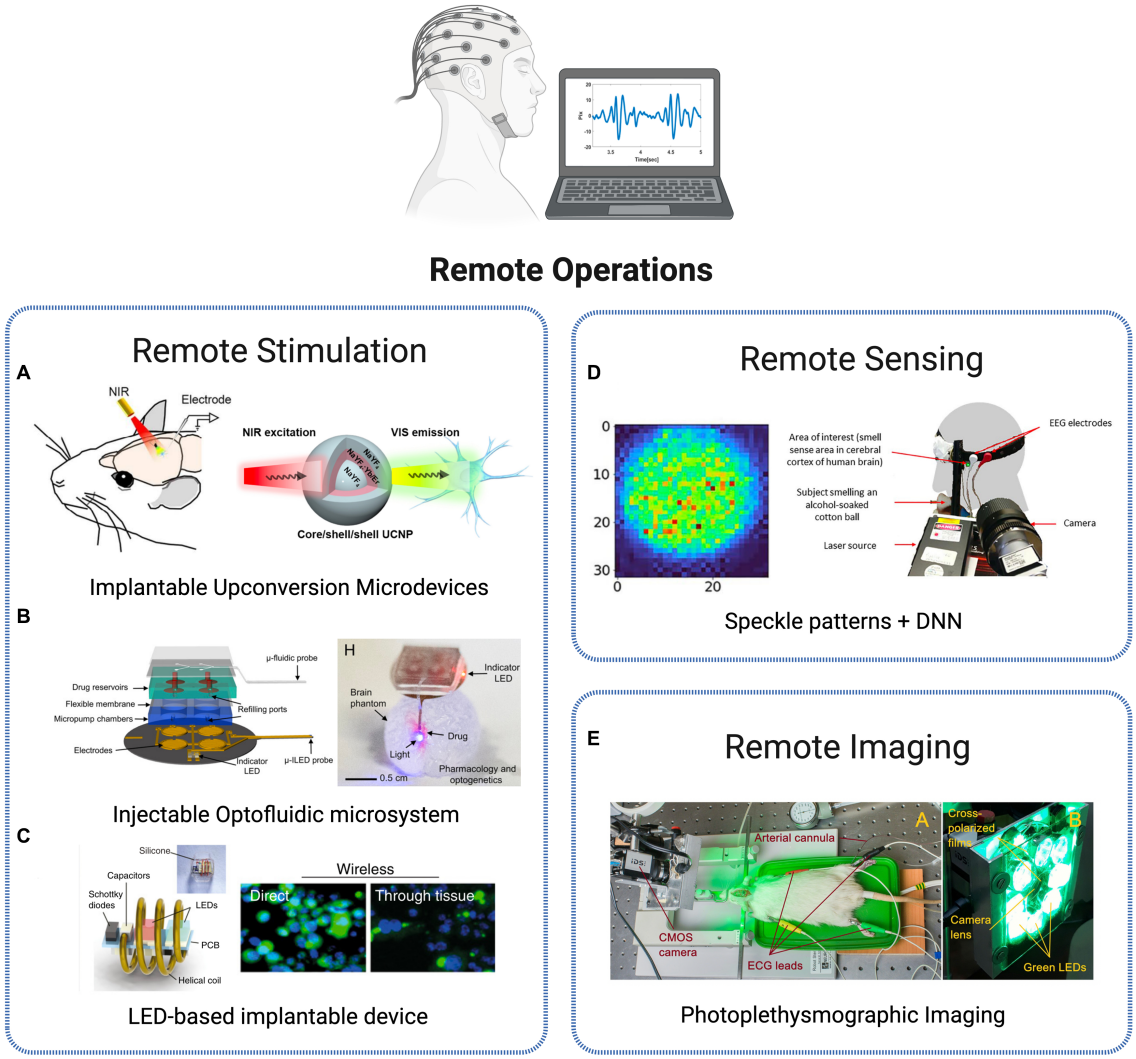
Strategies for remote monitoring of brain activity. **(A)** UCNPs for neuronal inhibition with recordings of spiking activities in neurons expressing *eNpHR* proteins (Lin et al., 2018). Reprinted with permission from Lin et al. (2018). Copyright 2018 American Chemical Society. **(B)** Injectable, battery-free, wireless microsystem for optical stimulation and pharmacology via programmable microfluidic fluid delivery. Reproduced from Zhang et al. (2019). **(C)** Wireless device for *in vivo* photodynamic therapy of tumor cells reproduced from Bansal et al. (2018). **(D)** Remote detection of human senses via synchronized recording of speckle patterns and EEG signal (Kalyuzhner et al., 2022). Licensed under CC BY 4.0. **(E)** Cerebral autoregulation monitoring via contactless green-light imaging photoplethysmography (Lyubashina et al., 2019). Licensed under CC BY 4.0.

In optogenetics experiments, light is typically delivered through implanted optic fibers that are connected to a light source. However, a considerable limitation of tethered systems is the inherent restriction of experimental designs and movements of the animals under study. To address this issue, tether-free optogenetic strategies have been suggested, for both remote stimulation and sensing applications. Examples are presented in Figure 7.

An all-optical approach is suggested in Wang et al. (2017) and Lin et al. (2018) using the previously introduced (Section IIIA) upconversion materials. The group presents a fully implantable micro-device with incorporated NaYF4-based upconversion nanoparticles. In Wang et al. (2017), remotely applied NIR illumination leads to a successful activation of spiking activity in rat brains (striatum, ventral tegmental area, and visual cortex). The microscale device was activated using a NIR laser at a wavelength of 980 nm, causing it to emit green or blue light, contingent on the dopants that were present in the nanoparticles. Upon implanting the optrodes in the brain infected with ChR2 or C1V1 to express themselves, the neurons could be reliably stimulated to generate action potentials by the NIR illumination. The results show successful conditioning of motor behavior in freely moving animals. A similar result was obtained with the same type of device for neural inhibition, using light to activate the commonly used inhibitory opsin protein Chr (Lin et al., 2018). By implanting the device in specific regions deep inside the rat brain and using near-infrared irradiation, the electrical activity of neurons can be consistently blocked, and it returns to its normal level once the NIR light is turned off (Figure 7A). This system was also tested in mice to inhibit the secondary motor cortex without any tethering, allowing for control of the animal motor functions. In Jeong et al. (2015), the authors present a multifunctional, innovative wireless neural probe that allows for the delivery of agents and optical manipulation in deep brain tissue of freely behaving animals. These probes use ultrathin and flexible microfluidic technology for drug delivery, along with micro–Inorganic Light-Emitting Diode (μ-ILED) arrays that can operate at the cellular scale. They offer the possibility to control the spatiotemporal delivery of fluids and photostimulation wirelessly, with a considerable reduction in size compared to the commonly used light-delivery cannulas. These probes have been tested in animals and have been shown to modify gene expression, deliver peptide ligands, and manipulate reward-related behavior in the mesoaccumbens region through concurrent photostimulation and antagonist drug delivery. A more advanced design by the same group was recently presented (Zhang et al., 2019), offering additional features such as the ability to refill drugs and programmable control of the flow rate. The completely wireless, battery-free and lightweight device is also capable of operating independently in various modes by infusing different types of drugs or delivering optical stimulation (Figure 7B). Moreover, the device’s design is compatible with current large-scale manufacturing methods, highlighting its potential for widespread adoption within the neuroscience community.

Remote operation was also demonstrated for therapeutic applications. In order to improve the compatibility and light delivery capabilities of photodynamic therapy technique, authors in Bansal et al. (2018) developed an innovative wireless photonic approach using an implantable miniaturized device (Figure 7C). In photodynamic therapy, optical irradiation triggers the activation of light-sensitive drugs, also known as photosensitizers. The process results in the selective elimination of cancerous cells without the negative side effects commonly associated with systemic treatments like chemotherapy. The approach suggested consists of wireless delivery of light into the brain, activating photosensitizers (Chlorin e6) through thick (>3 cm) tissues, inaccessible by direct illumination. This way, multiple controlled light doses are efficiently delivered for tumor growth suppression *in vivo*.

Recently, a novel paradigm in photonic sensors has been introduced with the concept of remote photonic sensing techniques to assess neural activity. This approach involves the analysis of spatiotemporal back-scattered light to sense sound waves of brain blood vessels (Ozana et al., 2020). It has been used in the development of various biomedical applications, such as heart rate monitoring (Zalevsky et al., 2009), blood coagulation, pressure and oximetry measurements (Ozana et al., 2015; Golberg et al., 2018; Kalyuzhner et al., 2021) and melanoma detection (Ozana et al., 2016). A novel approach to remotely monitor human brain activation is presented by utilizing the detection of task-related hemodynamic changes (Ozana et al., 2020). Physiological processes linked to neural activity, such as nano-vibrations caused by blood flow and tissue oxygenation in the brain, are identified through remote sensing of nano acoustic vibrations using temporal and spatial analysis of defocused self-interference random patterns, enabling remote real-time monitoring of neural activity. A new photonics-based remote sensing method for detecting human senses is proposed, combining deep learning with spatiotemporal analysis of defocused self-interference random speckle patterns reflected from the temple area of the human head (Kalyuzhner et al., 2022). The method projects a laser beam onto the specific area of the human head associated with cerebral cortex activity and analyzes the recorded speckle patterns using Deep Neural Networks (DNN) for the detection of human senses (Figure 7D). More advanced paradigms have been suggested, such as the Neurophotonic Solution-dispersible Wireless Activity Reporter for Massively Multiplexed Measurements (Neuro-SWARM3) system-on-a-nanoparticle probe solution presented in Hardy et al. (2021), enabling wireless detection of bioelectric signals with single neuron resolution. Using near-infrared light, the platform allows *in vivo* electrophysiological activity detection through the conversion of bioelectric field oscillations to optically detectable signal that can be captured from outside the brain, merging in a single nanoparticle signal detection and data broadcasting capabilities with wireless power transfer.

Another major group of remote sensor technology is based on non-invasive optical imaging techniques to monitor brain blood volume changes in the blood vessels, by measuring several cerebral oxygenation parameters. The sensor’s probe is usually constituted by a light emitting component that transmits it to the living tissue and a photoreceiver to acquire the backscattered light that is used to derive the physiological parameters. Such optical modalities, such as Laser Speckle Contrast Imaging (LCIS), PPG, NIRS and new derivate techniques such as speckle contrast optical spectroscopy (SCOS) constitute the basis of monitoring blood flow parameters in Neuroscience case studies (Liu et al., 2023; Konovalov et al., 2024). Lyubashina et al. (2019) present a contactless green-light imaging PPG method to assess spatial temporal variations of cerebral blood pulsations (Figure 7E). The custom-made PPG system was built using a digital CMOS camera combined with an illuminator, surrounded by a set of LEDs generating incoherent light generated, linearly polarized. This system was used in combination with electrocardiogram and systemic arterial blood pressure monitoring, to evaluate the open parietal cortex of male Wistar rats before, during and after visceral or somatic stimulation. The PPG method showed high suitability to assess alterations of cerebrovascular regulation mechanisms in response to physiological events where occur dynamic changes in the cortex blood supply to the cortex. Therefore, this represents a non-invasive, contactless and cost-efficient optical technique that can have a tremendous value, for instance, in neurosurgical intervention settings, to monitor viability of the cortex vessels and determine the state of patient’s cerebrovascular autoregulation.

### Microfluidic technology

**Figure 8:**
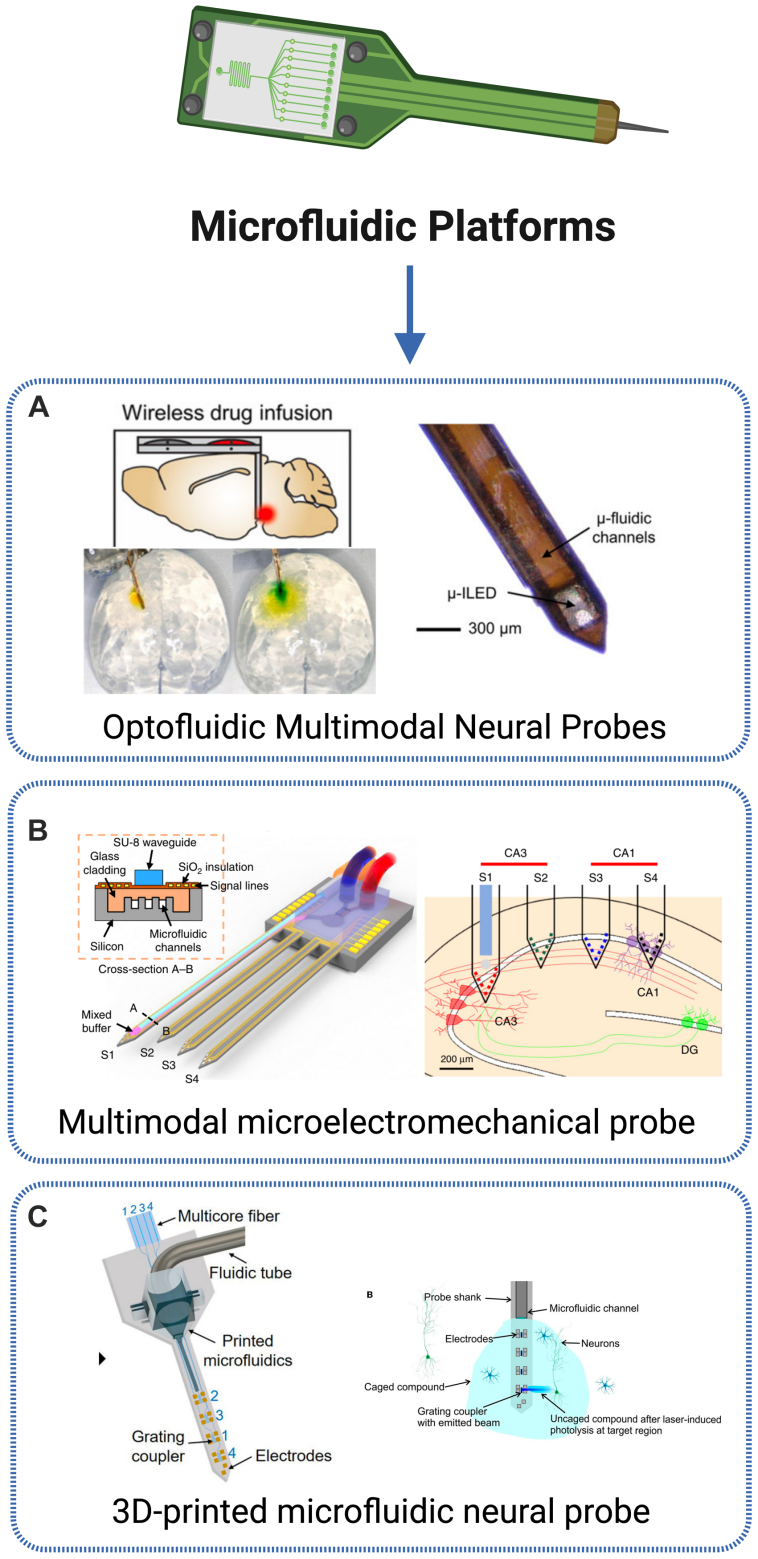
Microfluidic technologies. **(A)** Optofluidic probe for *in vivo* wireless delivery of fluidic compounds and optogenetic stimulation of neurons (Zhang et al., 2019). **(B)** Multifunctional multi-shank MEMS neural probe with neural recording and optical stimulation to investigate brain connectivity between areas of the hippocampus of a transgenic Thy1-ChR2-YFP mice (Shin et al., 2019). **(C)** Photonic neural probe with 3D printing integration of microfluidic channels. The probe allows for simultaneous optogenetic stimulation, extracellular recording and fluid injection, as demonstrated through an uncaging experiment (Mu et al., 2023). Created with BioRender.com.

The synergy of microfluidics and Neurophotonics holds the promise of unlocking deeper insights into brain function and neurological disorders, fostering advancements in both fundamental neuroscience research and potential therapeutic interventions. The use of miniaturized systems, applying the principles of microfluidics and photonics, offers transformative approaches to studying neural processes and conducting experiments with unprecedented control and sensitivity. An example of such combined approach relies on the efforts for integrating microfluidic channels into neural probes for several applications, from fluid injection and sampling of extracellular fluids to delivery of pharmacological agents to local targeted deep brain regions. Examples of such type of multimodal platforms were presented in Figure 8. In Jeong et al. (2015) and Zhang et al. (2019) authors developed an advanced wireless, lightweight and reusable optofluidic device suitable for pharmacological experiments through ultrathin and flexible microfluidic technology for drug delivery, while simultaneously allowing optogenetic interrogation using μ-ILED arrays that can operate at the cellular scale (Figure 8A). The characteristics and design of the device show high suitability for experiments *in vivo* experiments in freely moving mice. Beyond the microfluidic and optogenetic operations, Shin et al. (2019) reports a multimodal microelectromechanical system neural probe with additional electrophysiological recording functions. The multi-shank structure design allows to integrate chemical delivery of drugs in the deep brain, while simultaneously using optical stimulation (optical waveguide) for long-range neural circuits modulation (hippocampal CA3 – CA1 regions) and neural activity recording (32 electrodes), showing a successful integration of microfluidics with the most common functions of multimodal neural interfaces (Figure 8B). Another example of such successful integration is provided by Mu et al. where additive two photon polymerization 3D printing technology is suggested as a flexible, highly customizable method of integrating microfluidic structures onto neural probes (Figure 8C), since microfluidic channels can be directly printed onto neural probes of different designs, offering a high degree of flexibility in the manufacturing process (Mu et al., 2023). Through an uncaging experiment, where caged fluorescein is injected in brain tissues through the 3D-printed microfluidic channels of the neural probe, while light beams are selectively activated to uncage small sections of the fluorescein bolus, authors demonstrate the potentialities of integration in the same platform neurochemical compounds injection with photonic and electrophysiological capabilities.

## Discussion and outlook

Several marks in the history of science show the remarkable advancements possible when different scientific fields are combined to tackle common goals. The cross-disciplinary field of neurophotonics introduced in this work provides an excellent example. The integration of traditional neuroscience methodologies with light-based control techniques applied in neurophotonics field has resulted in the development of innovative tools for various purposes, spanning from fundamental studies in neuroscience and neurobiology to clinical diagnostics, functional imaging, brain-computer interfaces, and more. The neurophotonic revolution has been made possible by the convergence of key optical and genetic breakthroughs in the past 25 years. Optogenetics has revolutionized neuroscience by providing a toolkit for manipulating neuronal circuits using light, allowing for individual and non-invasive readout and control of the activity and signaling of multiple brain cells and synapses. Genetically encoded tools that can monitor and alter neural activity have expanded the capabilities of neuroscientists, enabling not only observation of neural activity but also precise perturbation with unprecedented ease. Recent progress in the development of sensors, such as enzymatic and neurotransmitter sensors, and the use of fiber-optic probes for selective light delivery and collection in deep brain regions, has created unparalleled possibilities to connect complex cellular signaling with behavior, spanning from individual cell photometry to recordings from extensive cell populations. The ability to simultaneously detect neuronal activation in hundreds or even thousands of cells non-invasively has been a game-changer in understanding how networks of brain cells function and interact dynamically, both in laboratory settings (*in vitro*) and in living organisms (*in vivo*). In this systematic review, a wide perspective on the last 10 years of research in Neurophotonics is provided, by analyzing and discussing innovative photonic approaches for Advanced optogenetics, Multimodal neural interfaces, Imaging devices and probes, Targeted therapeutics, Remote operations and microfluidic platforms. The innovation along this timeframe is remarkable and shows how rapidly this field is advancing. The main challenges faced across all areas discussed and the corresponding solutions identified are presented in Table 3.

**Table 3:** Main challenges identified across the 5 areas analyzed (Advanced optogenetics, Multimodal neural interfaces, Imaging devices and probes, Targeted therapeutics, Remote operations and Microfluidic platforms).

Challenges	Solutions
Lack of cell-type specificity	Patterned stimulation Multi-site stimulation capabilities Nanoscale precision via nanophotonics (Magnetic NPs, TNPs) Nanoprobes with sub-millisecond temporal precision
Efficient and precise light delivery	Micro and nanofabricated multi-site light delivery probes Thin, flexible waveguides Upconversion NPs Paralell operations: probing multiple brain targets Embedded nanophotonic components (complex illumination patterns) Lightweight light-delivery strategies (Micro organic-LEDs)
Multifunctionality into a single platform	Miniaturized optrodes and fibertrodes Micro and nanofabrication techniques (3D laser lithography, 2P polymerization) Multiple light-delivery & electrical recording sites Ultracompact optoeletronic probes Addition of sensing capabilities Multifunctionality embedded into NPs Microfluidic channels into neural probes (drug-delivery)
High-resolution brain imaging	Photostable fluorescent proteins Photoactivation properties Miniaturized head-mounted microscopes Two-photon high-resolution imaging Implantable micro-imagers (fiber bundles, light sheets) *In-vivo* high-precision measurement technology (photometry, tomography) Low-cost, portable, and customizable designs Particle self-tracing properties
Minimal invasiveness and damage	General miniaturization of tools used to deliver, manipulate, and collect light Tapered, ultra-narrow & nanostructured fibers Biocompatible, bioresorbable, and biodegradable materials Flexible and small organic LEDs Wearable, tetherless and/or wireless approaches Micro and Nanofabrication techniques (Electron beam lithography, nanophotonic circuits, micro-arrays) Thermal and light upconversion materials (NIR light-responsive probes) Mechanically compliant platforms (miniature dimensions, low-bending stiffness) Nanoscopic platforms with multifunctional capabilities (Quantum sheets, TNPs) Polymer-based probes Flexible and compact implantable devices

Corresponding solutions to tackle such challenges, suggested in the articles covered in this work. NPs, Nanoparticles; LED, Light-Emitting Diode; 2P, Two-Photon; 3D, Three-dimensions; NIR, Near-Infrared light.

Since the first demonstration of laser stimulation in abdominal ganglion neurons (Fork, 1971), neuromodulation techniques are highly challenged by the lack of specificity for cell types. Remarkable advances in genetics and molecular biology enabled selective photostimulation of genetically modified neurons, offering continuous improvements in achieving cell-type specificity. Examples range from initial solutions using patterned optogenetic stimulation (Reutsky-Gefen et al., 2013) to more advanced technologies offering multi-site optogenetic stimulation capabilities using complex optical fiber geometries (Pisanello et al., 2015). For optogenetics in the deep brain, implantable photonic devices started to be developed, with complex light delivery capabilities (Segev et al., 2017). More recently, the ability to deliver light to specific cells or regions with nanoscale precision was introduced by probes with embedded nanophotonic components and the use of upconversion nanoparticles, allowing for highly targeted optogenetic stimulation (Chen et al., 2018). This advancement has expanded the reach of optogenetic stimulation to deeper brain structures, enhancing our understanding of the neural networks involved in various brain functions.

Along with cellular specificity, another major obstacle is the efficient delivery of light to neural tissues, deeply rooted in the intrinsic interaction between light and matter. To address this challenge, many methods have been discussed and developed to deliver light precisely to the targeted region in the nervous system. Early techniques for delivering light to deep tissues, such as fiber-based devices or large chip shank-like designs, are effective but have downsides as a consequence of the large cross-sections that can cause tissue damage, trigger an immune response, and disrupt the biological system (Williams et al., 2007). In response to the invasiveness issues of such traditional light delivery probes, innovative methods such as multi-photon stimulation, micro and nanofabricated multi-site light delivery probes (Nathan Perkins et al., 2018) and organic LEDs (Ciccone et al., 2022) have been presented, providing high levels of precision and resolution in delivering light to deep tissues while minimizing tissue damage and immune response. Additionally, as UCNPs possess the unique ability to convert NIR light that can penetrate the brain into visible emission, several research groups have shown successful deep-brain optogenetics by either implanting devices containing UCNPs or injecting UCNPs into the specific region of interest in the brain. Another major trend over the last years is the incorporation of multiple functionalities into a single platform, creating advanced multimodal neural interfaces. The first optrode, combining optogenetic manipulation with electrical readout of neural activity, was suggested in 2007 (Gradinaru et al., 2007), and since then, significant progress has been made in increasing the density of stimulation/readout points. Innovative approaches for investigating causal connections within brain microcircuits have been made possible by the emergence of new types of implantable nano- and microphotonic devices. One promising approach for multisite modulation involves implanting multiple solid-state light sources directly into the specific brain region(s) of interest. More advanced designs have emerged, featuring multiple sites for light delivery and electrical recording. In order to minimize invasiveness and tissue damage, the use of micro and nanofabrication techniques is becoming a popular approach, providing new opportunities to produce compact, integrated, and scalable optoelectronic probes. Besides, tapered fiber geometries are now suggested with fully integrated microelectrode arrays to combine multi-point light delivery with spatiotemporal recording capabilities (Pisanello et al., 2015). Additional to stimulation and recording operations, a new optical dimension has been explored in optical implantable devices, based on sensing physiological parameters such as temperature and tissue oxygenation within the brain (Bai et al., 2019).

Considerable technological advances have also been observed in imaging techniques, offering high spatial resolution. A pivotal advancement in enabling high-resolution, high-throughput imaging of labeled brain structures in animals is the development of a diverse array of bright, photostable fluorescent proteins. These innovations have allowed researchers to image deeper within living organisms, leading to a proliferation of strategies for manipulating and controlling light to enhance temporal resolution, photoactivation, and extended field-of-view imaging. Wearable imaging systems have been engineered to facilitate measurements in freely moving model organisms. These miniaturized microscopes come in various implementations, with different imaging capabilities, from single-photon fluorescence (Liberti et al., 2017) to two-photon high-resolution imaging (Zong et al., 2017), providing higher penetration depths into scattering tissue, that allow to cover a large field of view while retaining high resolution and speed. Recent advancements in the field of fiber-based tools have facilitated high-speed fluorescence microscopy in freely moving animals, overcoming long-standing challenges in studying the neuronal underpinnings of cognition and memory. These developments have resulted in a diverse range of implantable micro-imagers that are specifically designed for functional brain imaging with cell-type specificity, paving the way for enhanced experimental investigations in this area. Examples include the use of optical fiber bundles (Fedotov et al., 2017), light-sheet fluorescence or imaging devices applying photometry (Simone et al., 2018) and tomography (Orive-Miguel et al., 2020) to provide *in vivo*, high-precision measurements of calcium transients or oxy and deoxyhemoglobin tissue concentrations, respectively.

A challenge across all application areas of neurophotonic techniques is to develop methodologies with minimal invasiveness and damage to the tissues. Multiphoton stimulation and microfabricated multi-site light delivery probes have emerged as notable techniques in addressing invasiveness issues. Another approach is the use of tapered, ultranarrow, and nanostructured optical fibers (Spagnolo et al., 2022). These fibers offer high flexibility, small size, and high spatial resolution, allowing for controlled and precise light delivery within the brain tissue. These advanced fibers are being explored as a promising solution for achieving less invasive optical stimulation in deep neural tissue. In addition to addressing invasiveness challenges, the compatibility and stability of optical fibers within neural tissue have also been investigated. Common silica fibers may pose compatibility and stability issues, and thus, biocompatible and biodegradable materials have been explored as alternatives. Furthermore, OLEDs have been proposed as an alternative to overcome the rigidity and bulk design of commonly used LEDs. OLEDs offer flexibility and smaller form factors, making them suitable for minimally invasive operations within the brain while providing efficient light emission for stimulation or detection purposes (Ciccone et al., 2022). The switch to smaller and more flexible devices has also allowed to substitute commonly used tethered neural interfaces for wearable, tetherless and/or wireless approaches, thus reducing the restrain of animal’s native behaviors that would cause considerable constraints in experimental designs. In this context, another trending solution over recent years is the miniaturization of tools used to deliver, manipulate and collect light within deep brain regions. The combination of optoelectronic probes with microfluidic technologies is creating opportunities in the development of programmable drug-delivery platforms (Jeong et al., 2015). Nanofabrication techniques applied to optical probes and interfaces are continuously reported to integrate miniaturized components for multi-functionality capabilities. For instance, the use of upconversion nanoparticles (Zhou et al., 2020) is being highlighted as efficient approaches for highly specific light and drug delivery within the brain.

Advances in all aforementioned areas are a consequence of underlying technological progress regarding materials, electronics and system designs. A dominating theme in electrical interfaces is the development of mechanically compliant platforms, in the form of thin, soft substrates with low bending stiffnesses and ultra-miniaturized dimensions. The use of nano-structuring or mixed conduction mechanisms (ionic–electronic) in polymers and nanocomposites allows an increase in the electrochemical surface areas. In recent years, researchers have increasingly chosen a major approach involving the use of flexible probes that integrate electronic and optoelectronic components on thin and narrow polymer fibers at a cellular scale. The development and optimization of up-conversion materials are creating tremendous opportunities to develop nanoscopic multifunctional platforms. Wireless devices, especially those with fully implantable with battery-free designs, are allowing optogenetics, photometry, oximetry, pharmacological delivery, temperature monitoring and even measuring of vascular blood flow for large periods of time with minimal acute and chronic damage. To deal with foreign-body response, biocompatible and bioresorbable materials, as well as mechanically compliant neurointerfaces are crucial to overcome the biological barrier associated with chronic implants.

## Future challenges

The rise of innovative opportunities is followed by a vast panoply of new challenges. The comprehensive characterization of even simple brain circuits at the single-neuron level remains hampered by the lack of a robust system for fast and accurate reconstruction of neuronal branching structures. Although numerous neurons have been digitized across a wide range of brain regions, variability introduced by differences in species, brain location and methodology has made systematic analysis and comparison difficult. In a broad-spectrum analysis, we highlight three main challenges for innovation and translation in Neurophotonics: Light delivery in the deep brain, physical constraints, and data management.

Firstly, as already discussed along this review, an effective delivery of light within the brain remains one of the greatest challenges in the field of Neurophotonics. Due to the presence of a vast arrangement of biological structures, from endogenous chromophores or lipids in a wide range of size scales (e.g., neuron membranes and the myelin sheath), neural tissues are a highly scattering and absorbing medium which challenges efficient and precise optical targeting tasks, mainly due to problems related to light distortion and aberrations. Overcoming these challenges has been the driving force behind innovative technological solutions proposed, developed and tested, from multi-photon microscopy to advanced neural probes for deep-brain optogenetics. Promising approaches to achieve more precise and efficient light delivery are centered on the use of novel light sources based on upconversion materials or infrared-to-visible converters, which can convert deep-penetrating near-infrared light into visible light that can be absorbed by neural cells. The integration of such property with carriers of small size, such as nanoparticles, allows delivery of light with minimal acute and chronic damage to the tissue and reduced restraint of the animal’s native behavior. Another approach is the use of wavefront shaping techniques to control the scattering of light within the brain, allowing for more precise and efficient targeting of neural cells. Additionally, the development of advanced imaging and sensing techniques, such as adaptive optics and optogenetic sensors, can help to overcome limitations in spatial resolution and sensitivity, enabling more accurate and detailed measurements of neural activity in deep brain regions.

Secondly, the overall development of new materials and techniques for optical neural interfacing must account for fundamental physical constraints. As previously discussed, the interaction between light and biological tissues imposes restrictions on the penetration depth and allowable power density, which limit the use of light-based methods in larger-brained organisms, including humans, without invasive interventions. The insertion of optical devices into deep brain regions is a challenging task due to their low cross-sectional sharpness and invasiveness. When considering photonic implants, designs should integrate multiple functionalities while minimizing its profile, to mitigate damage to brain tissue. Besides, in order to ensure applicability in unrestrained animal models, optical methodologies must be compatible with such experimental settings. To enable this, it is essential to devise integrated wireless systems that facilitate spatiotemporal control of multiple stimuli, including light and drug delivery, and offer storage for onboard recorded signals for post-processing analysis. Experiments involving freely moving animals will strongly benefit from the development of wireless power systems to mitigate the issues of weight and unreliability associated with onboard batteries. Additionally, the development of mechanically compliant neurointerfaces and biocompatible materials, with long-term stability, is crucial to deal with the barrier imposed by the foreign-body response. We envision that the miniaturization of devices and processes will continue to be one of the most notable trends in Neurophotonics research. The primary advantage of miniaturization in Neurophotonics is the potential for increased precision and selectivity in targeting specific regions of the brain. Smaller optical devices enable more precise spatial resolution and can be more effectively targeted toward specific cell types. Additionally, miniaturized devices can reduce tissue damage and inflammation, and minimize the impact on surrounding brain tissue. Another significant advantage is the potential for increased portability and ease of use since smaller devices can be more easily integrated into experimental setups and could potentially facilitate *in vivo* imaging and manipulation in freely behaving animals. One very interesting example is the use of tissue chip (TC) devices, designed to imitate human physiology on a small scale, which present an opportunity to improve upon traditional animal models in terms of reproducibility, human relevance, and ethical and monetary cost savings. When combined with the sensitivity of photonic sensors such as ring resonators, it becomes possible to perform label-free, highly sensitive detection of cellular responses, as already demonstrated for the detection of inflammatory cytokines into a TC model (Cognetti et al., 2023). This represents the first application of photonic sensors in a human TC device and opens the door to new possibilities in drug development and disease modeling through miniaturized, portable devices.

Despite the exciting potentialities, some important challenges must be overcome. One significant limitation is the trade-off between device size and performance. As devices become smaller, there is a risk of decreased signal-to-noise ratio, light output and photon collection efficiency and overall sensitivity. Smaller devices may be less suitable for imaging or manipulation in deep brain structures, due to decreased penetration depth and signal quality. Overcoming this inherent challenge requires a collaborative effort among experts in material science, optical, and photonics engineering to map and consider the physical principles governing neuromodulation when designing new optical neural interfaces with smaller footprints and higher throughput. Next generation technology will need to prioritize the development of neuronal interfaces with multifunctional capabilities, capable of targeting both small and large brain volumes. The advancement of implantable devices has paved the way for the development of optical neural interfaces that can access multiple points of the brain simultaneously. To fully realize their potential, it is now necessary to integrate multiple functionalities into a single device. This will enable simultaneous control and monitoring of neural activity in various brain regions with customized spatial configurations for specific experimental needs. While current work has focused on multipoint light delivery, new techniques are needed for light collection and multi-point monitoring of neural activity, especially in deep-brain structures. Such interfaces could enable cell-type-specific modulation and readout of neural activity, allowing for powerful new approaches like closed-loop optogenetics. Moreover, non-optical components like microfluidics and integrated electrodes could complement these interfaces. For instance, microfluidics could be used for *in situ* drug delivery, while integrated electrodes could provide access to neuronal signals that cannot be measured optically, such as local field potentials.

Lastly, an important challenge that is starting to be explored is related to the managing, storage and interpretation of the large volumes of data generated by the currently applied methodologies. Given the brain’s intrinsic structural complexity, imaging resolution and depth are continuously pushed to their limits while researchers attempt to cover increasingly large areas, in order to achieve a system-level understanding of this organ. This results in massive data acquisition and processing needs. To further develop the field of Neurophotonics, efficient methods for storing the vast amounts of data generated from opto-electrical signal and image recordings must be found. The rising demand for these techniques in challenging situations is spurring, in recent years, the development of novel data analysis methodologies and algorithms that enhance accuracy, depth penetration, and spatial sensitivity. The application of computational techniques to optical engineering in the nervous system has the potential to improve the ability to penetrate turbid media. Future advancements in computational techniques for correcting light scattering through a combination of computational and optical methods may play a role in imaging deep into turbid brain tissue. Computational algorithms have shown tremendous success in performing signal extraction tasks, such as segmentation, image deconvolution or spike estimation. Most data analysis methods are still dependent on the time-consuming task of manual identification and labeling of regions-of-interest, an area where automated image analysis computational approaches can be a valuable contribution. Data-driven machine/deep learning-based techniques are becoming popular, achieving state-of-the-art performance in various tasks, such as vessel and neuron segmentation in deep brain (Stefan and Lee, 2020; Bao et al., 2021), 3D deconvolution (Wang et al., 2021), and signal denoising (Lecoq et al., 2021). Besides, the use of computational algorithms is showing a tremendous potential to help retrieve more information from data collected using well-stablished techniques, by improving its quality, allowing the removal of important artifacts or by deriving new parameters and insights from large amounts of raw data. Examples include the spreading adoption of AI to improve blood flow imaging using LSCI images (Yu et al., 2023) or the crescent application of deep-learning algorithms in the analysis and automated feature extraction of PPG-derived data, overall enabling more data-efficient and reliable signal extraction (Yen et al., 2022). In the future, computational microscopy will likely continue to affect system design and signal analysis in neuronal imaging. One promising approach is the joint optimization of optics, acquisition strategy, and computational algorithms in a holistic framework to push imaging performance limits further. To fully understand the complex network of cells in the nervous system, next-generation technologies must be able to communicate with neural tissue through a variety of modalities, timescales, and sensitivities at scales ranging from nanometers to centimeters. Fabrication techniques and materials must evolve to accommodate novel optical and chemical probes for use in live subjects and allow continuous recording of multiple biomarkers. However, increasing experimental complexity can hinder data interpretation, and care must be taken when selecting tools to avoid confounding artifacts and unsupported conclusions. Correct use of these tools will enable studies of the molecular mechanisms underlying behavioral and physiological phenotypes and facilitate the integration of molecular and systems neuroscience. This integration will inform therapeutic approaches for conditions with heterogeneous pathophysiology and evolving signatures. The development of image processing and analysis tools will enable the creation of new brain atlases and a better understanding of the connectome. This may facilitate the study of atrophies in brain diseases and identify new targets for neuromodulation treatments. This presents unlimited research opportunities for biologists and engineers and should motivate the translation of these emerging approaches and insights from the laboratory to the clinic environment.

## Author contributions

BB: Conceptualization, Investigation, Writing – original draft, Writing – review & editing, Data curation, Methodology. JC: Conceptualization, Formal analysis, Funding acquisition, Investigation, Project administration, Resources, Supervision, Validation, Visualization, Writing – original draft, Writing – review & editing.
